# HF‐Free Boc Synthesis of Peptide Thioesters for Ligation and Cyclization

**DOI:** 10.1002/anie.201607657

**Published:** 2016-10-06

**Authors:** Richard Raz, Fabienne Burlina, Mohamed Ismail, Julian Downward, Jiejin Li, Stephen J. Smerdon, Martin Quibell, Peter D. White, John Offer

**Affiliations:** ^1^ The Francis Crick Institute1 Midland roadLondonNW1 1ATUK; ^2^ Sorbonne Universités, UPMC Univ Paris 06, ENS, CNRSLaboratoire des Biomolécules (LBM)ParisFrance; ^3^ Département de Chimie, ENSPSL Research University, UPMC, Univ Paris 06, CNRS, LBMParisFrance; ^4^ Merck ChemicalsPadge Road, BeestonNottsNG9 2JRUK

**Keywords:** Boc-SPPS, cyclic peptide, peptide ligation, peptide thioester, post-translational modification

## Abstract

We have developed a convenient method for the direct synthesis of peptide thioesters, versatile intermediates for peptide ligation and cyclic peptide synthesis. The technology uses a modified Boc SPPS strategy that avoids the use of anhydrous HF. Boc in situ neutralization protocols are used in combination with Merrifield hydroxymethyl resin and TFA/TMSBr cleavage. Avoiding HF extends the scope of Boc SPPS to post‐translational modifications that are compatible with the milder cleavage conditions, demonstrated here with the synthesis of the phosphorylated protein CHK2. Peptide thioesters give easy, direct, access to cyclic peptides, illustrated by the synthesis of cyclorasin, a KRAS inhibitor.

Peptide thioesters are key precursors for the synthesis of proteins^1^ and cyclic peptides^2^ (Scheme 1). The demand for peptide thioesters has increased with the success of native chemical ligation (NCL). One of the greatest obstacles to using NCL is the challenging synthesis of peptide thioesters.

**Scheme 1:**
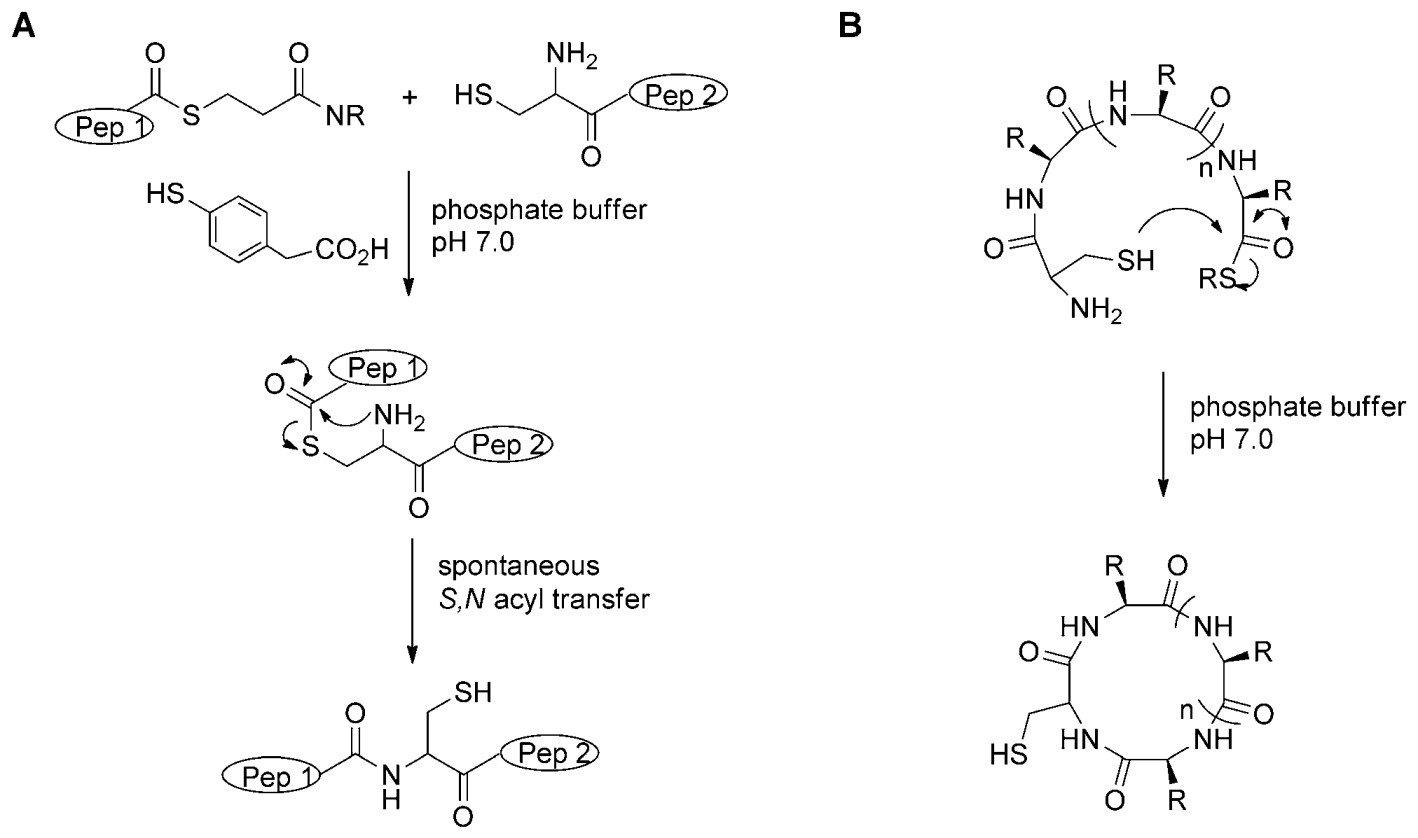
Applications of peptide thioesters. A) Native chemical ligation for the coupling of peptides and proteins by linking two unprotected segments, one a peptide thioester. B) Head‐to‐tail cyclization of peptides containing both an N‐terminal cysteine and a C‐terminal thioester.

Initially, peptide thioesters were prepared directly by *tert*‐butyloxycarbonyl (Boc) solid phase peptide synthesis (SPPS) facilitated by the stability of the thioester bond to trifluoroacetic acid (TFA), used for Boc deprotection cycles.^3^ The major limitation of Boc SPPS is its requirement for anhydrous HF for the deprotection and cleavage of the peptide from the resin. HF requires specialized apparatus and training.^4^ This has restricted Boc SPPS to a few laboratories experienced with handling this extremely hazardous reagent.^5^ In addition, HF is not compatible with the incorporation of many post‐translational modifications. 9‐Fluorenylmethoxycarbonyl (Fmoc) SPPS has become the method of choice for the routine synthesis of peptides in a large part due to its avoidance of HF.^6^ There has consequently been a considerable effort to develop robust Fmoc SPPS methods for the synthesis of peptide thioesters.

The direct synthesis of peptide thioesters by Fmoc SPPS is complicated by the reactivity of the thioester bond. Coupling a fully protected peptide fragment to a thiol in solution, followed by deprotection remains a popular approach.^7^ Additionally, many ingenious, indirect approaches have been developed. There are two types: safety catch, examples of which include sulfonamide,^8^ *N*‐acylurea,^9^ hydrazide/azide;^10^ and acyl shift, either *O*,*S* 11 or *N*,*S*.^12^

Nevertheless, Boc SPPS possesses a number of advantages compared to Fmoc SPPS: higher solubility of Boc amino acids promotes faster coupling;^13^ TFA fully solvates the peptide–resin at each deprotection cycle, preventing peptide–resin aggregation and enabling the synthesis of long peptides;^14^ in comparison to Fmoc SPPS, there is much less aspartimide formation.^15^ In addition Boc deprotection is always complete, in contrast to Fmoc where partial deprotection during aggregation is problematic. Because of these attendant benefits a combination of Boc SPPS and native chemical ligation (NCL) has enabled the synthesis of many proteins of sufficient quality to be crystallized for structural studies.^16^

We therefore decided to explore a modified Boc SPPS route to peptide thioesters that used a milder acid for final cleavage. This approach would have the following benefits: the direct synthesis of peptide thioesters with a single, safer, cleavage step; the possibility of parallel synthesis and small‐scale cleavages and greater compatibility with post‐translational modifications. The superior quality of peptides synthesised by Boc SPPS was the main factor.^13^, ^17^

There have been many attempts to substitute HF with trifluoromethanesulfonic acid (TFMSA).^18^ However, in contrast to HF, it is difficult to remove as it is not volatile, and residual reagent can cause degradation of the peptide product. A two‐step deprotection has been evolved to address this problem.^19^

TFA/TMSBr (trimethylsilyl bromide) mixtures can cleave benzyl‐based side‐chain protection from Asp/Glu/Ser/Thr/Lys/Tyr/Cys and Mts from arginine.^20^ As demonstrated in the elegant work of the Yajima group TFA/TMSBr has many notable properties as a cleavage reagent: it is volatile, more so than TFA, and therefore easily removed by sparging; it reduces any methionine sulfoxide formed during synthesis; and benzyl deprotection of aspartyl residues is not accompanied by aspartimide formation.^20^ It is routinely used in Fmoc SPPS when stronger deprotection conditions are required.^21^

Unfortunately, TFA/TMSBr is not a sufficiently strong acid to achieve complete peptide cleavage from methylbenzylhydrylamine (MBHA) and 4‐(hydroxymethyl)phenylacetamidomethyl (PAM) resins and deprotect all the standard side‐chain protection of classical Boc SPPS. However, these limitations could potentially be overcome by combining TFA/TMSBr cleavage with Merrifield hydroxymethyl resin, Boc in situ neutralization protocols^14^ and changing some of the side‐chain protection for that identified by the Yajima group as most compatible: Arg to Mts, Cys to Mob and Merrifield's original choice of benzyl for Asp and Glu (Table S1 in the Supporting Information (SI)). Cyclohexyl had been introduced primarily because of concerns over aspartimide formation during HF cleavage with benzyl protection,15b not so problematic with TFA/TMSBr cleavage.^20^

The use of Merrifield resin for Boc SPPS was largely abandoned when 4‐(Hydroxymethyl)phenylacetamidomethyl (PAM) and 4‐methylbenzylhydrylamine (MBHA) resins were introduced, primarily because of reported peptide cleavage by TFA over the prolonged (20 min) TFA cleavage cycles.^22^ However, contemporary Boc SPPS in situ neutralization protocols with their shorter, typically, 2×1 min treatment with TFA per cycle^14^ are more suitable. Merrifield resin had also been associated with other side reactions, notably capping by trifluoroacetylation and formation of deletion sequences caused by the presence of aldehyde impurities on the resin.^23^ However, these problems were identified many years ago, before improvements in the chemical purity of commercial resins and before the adoption of HBTU and other uronium coupling agents that do not couple trifluoroacetic acid. Consequently, a re‐examination of Boc SPPS on modern preparations of Merrifield resin, with in situ neutralization protocols was timely.

First, the stability of Merrifield resin linked peptides to TFA was reinvestigated. For this, we used hydroxymethyl resin derivatized with Fmoc‐Gly because Gly is one of the more acid labile residues^22^ and Fmoc provides a good reporter to monitor loss. The rate of Fmoc‐Gly cleavage by neat TFA was monitored directly by following the release of Fmoc by UV (Figure 1 B). Less than 5 % amino acid cleavage from the resin was observed after 500 min TFA treatment. The experiment was repeated with Fmoc‐Gly attached to mercaptopropionicacidleucine (MPAL) linker (Figure 1 B) used for thioester synthesis. Chain loss was considerably slower with the MPAL linker presumably because of the comparably more hindered terminal leucine residue. In both cases the loss of amino acid was in general agreement with previous measurements.^22^ Although there was chain loss with the MPAL linker, at 1 % over 500 min, it was suitable for in situ neutralization cycles (Figure 1 B).

**Figure 1:**
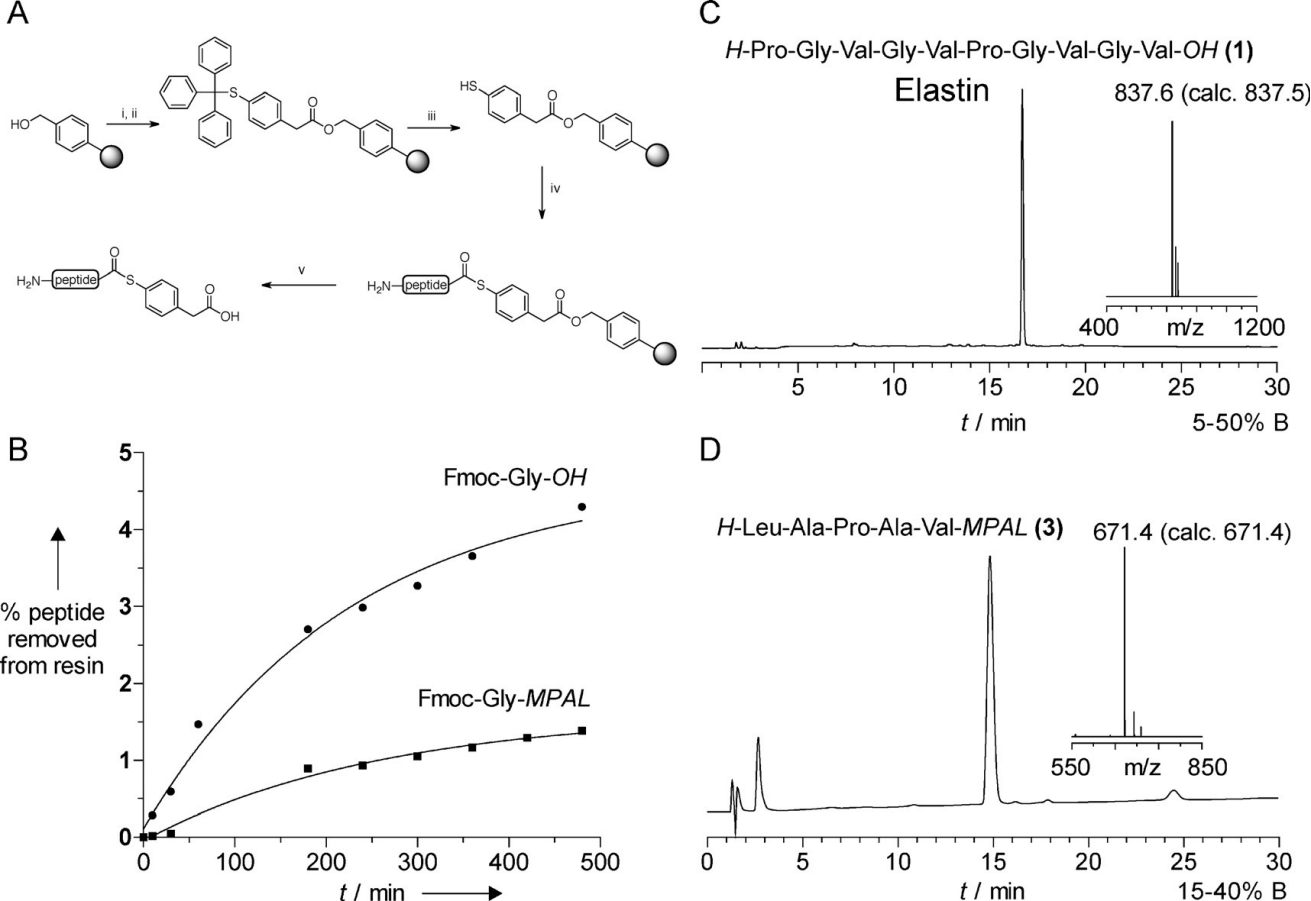
A) Direct synthesis of peptide thioesters by a modified Boc protocol illustrated for MPAA thioester. i) PBr_3_/DCM; ii) MPAA/DIEA/DMF; iii) TFA/TES/H_2_O; iv) Boc SPPS; v) TFA/TMSBr/thioanisole/EDT (1:0.05:0.05:0.025), 1 h. DCM=dichloromethane, DIEA=diisopropylethylamine, DMF=dimethylformamide. B) Time course of Fmoc‐Gly‐OH and Fmoc‐Gly‐MPAL release from Merrifield resin with TFA, monitored by Fmoc absorbance at 301 nm. C) Analytical HPLC and MALDI‐TOF MS of crude elastin synthesized with in situ neutralization Boc cycles on hydroxymethyl resin. D) Analytical HPLC and MALDI‐TOF MS of crude peptide thioester LAPAV‐MPAL.

Next, we investigated the use of Boc in situ neutralization cycles on Merrifield resin to see if the notorious capping and deletion reactions occur under these conditions. Elastin **1** (Table 1) has been synthesised recently by Kent and co‐workers and gave us a direct comparison of yield and purity to current, optimized, Boc‐SPPS.^24^ The resin was derivatized with PBr_3_ and Fmoc‐valine to obviate racemization.^25^ Synthesis was carried out manually with in situ neutralization Boc cycles and the peptide cleaved with TFA/TMSBr/thioanisole/EDT (1:0.05:0.05:0.025) at room temperature for 1 h. The cleavage mixture was sparged under a stream of nitrogen and the peptide precipitated from ice‐cold ether. No further peptide was recovered after a repeat cleavage of the resin. This simple, safe procedure contrasted to the lengthy HF cleavage and represented a considerable time saving. Success was reflected in the impressive final isolated yield 81 %, comparable with 61 % of Dang et al.^24^ Inspection of the analytical HPLC of the crude peptide (Figure 1 C) revealed no deletion sequences or capping. The high yield and simplicity of cleavage encouraged us to explore a more challenging target.

**Table 1:** Yields of peptides synthesized via modified Boc SPPS.

Peptide	Sequence	Crude yield [%]^[a]^	Isolated yield [%]^[b]^
**1**	*H*‐PGVGPGVGV‐*OH*	98	81
**2**	*H*‐GCCSDPRCRYRCR‐*OH*	77	21
**3**	*H*‐LAPAV‐*MPAL*	92	54
**4**	*H*‐LAPAA‐*MPAL*	90	50
**5**	*H*‐LAPAG*‐MPAL*	86	22
**6**	*H*‐LYRAF*‐MPAL*	81	34
**7**	*H*‐LAPAG*‐MPAA*	68	16
**8**	*H*‐LAPAA*‐MPAA*	84	31
**9**	*H*‐LAPAQ*‐MPAA*	83	25
**10**	*H*‐LAPAV*‐MPAA*	89	36
**11**	*H*‐LAPAT*‐MPAA*	86	36
**12**	*H*‐LAPAW*‐MPAA*	83	26
**13**	*H*‐LYRAI*‐MPAA*	91	28
**14**	*H*‐LYRAL*‐MPAA*	82	23
**15**	*H*‐LETVS_p_TQELY‐*MPAA*	57	23
**16**	*H*‐LKAQADIYKA*‐MPAA*	66	24
**17**	*H*‐dAlaArgArgArgdNalArgPhe(4‐F) dNleGlnTrpThr‐*MPAA*	84	16
**18**	*H*‐CdYVYNTRSGWRWYT‐*MPAA*	88	28
**19**	*H*‐AEQH(DNP)KIVMETVPLKAQA DIYKA‐*MPAA*	51	14
**20**	*H*‐LEDLRQQLQQAEEALVAKQE LI‐*MPAA*	80	19
**21**	*H*‐LEDLRQQLQQAEEALVAKQELI DKL‐*MPAA*	68	13
**22**	*H*‐LEDLRQQLQQAEEALVAKQELI DKLKEEA‐*MPAA*	64	10

[a] Yield calculated from resin loading and weight of crude, unpurified peptide. [b] Isolated yields calculated on dried weight of purified peptide (including TFA salts).

We chose a well‐characterized test peptide, α‐conotoxin RgIA **2** (Table 1). Its synthesis has been described in detail.4a It features a challenging sequence, rich in Arg and Cys. We substituted the Tosyl protection of Arg with Mts and the Meb protection of Cys with Mob. The side‐chain protecting group selection is shown in Table S1 (SI). Analytical HPLC of the crude material compared well with that obtained from conventional Boc SPPS (Figure S1, SI).4a

Having proved the strategy with peptide acids we wanted to test peptide thioesters. Initially, model thioester peptides were prepared using standard MPAL linker with short test sequences. The use of a spacer residue such as leucine before mercaptopropionic acid is proven to increase the final yield,3a although it is also used without.19b Several examples were synthesized and gave products of satisfactory purity by inspection of HPLC (Figure 1 D) and yield (Table 1).

NCL often makes use of the more kinetically activated thiophenyl esters.^25^ They have been synthesized on‐resin with a mercaptophenylacetic acid (MPAA) linker with conventional Boc SPPS.^26^ A series of MPAA peptide thioesters were synthesized here using or procedure (Figure 1 A). The weight gain of the resin was recorded to measure any possible chain loss either from TFA lability or instability of the thioester bond to the coupling conditions (Table 1). The weight gain suggested that losses were low and the crude product were of generally excellent quality (Figure 2). The weight gain was a better guide than isolated yield as the latter was very variable depending on efficient loading of the linker, solubility of the peptide and the equipment used for purification. Examples were chosen to test compatibility with all the amino acids (Table 1).

**Figure 2:**
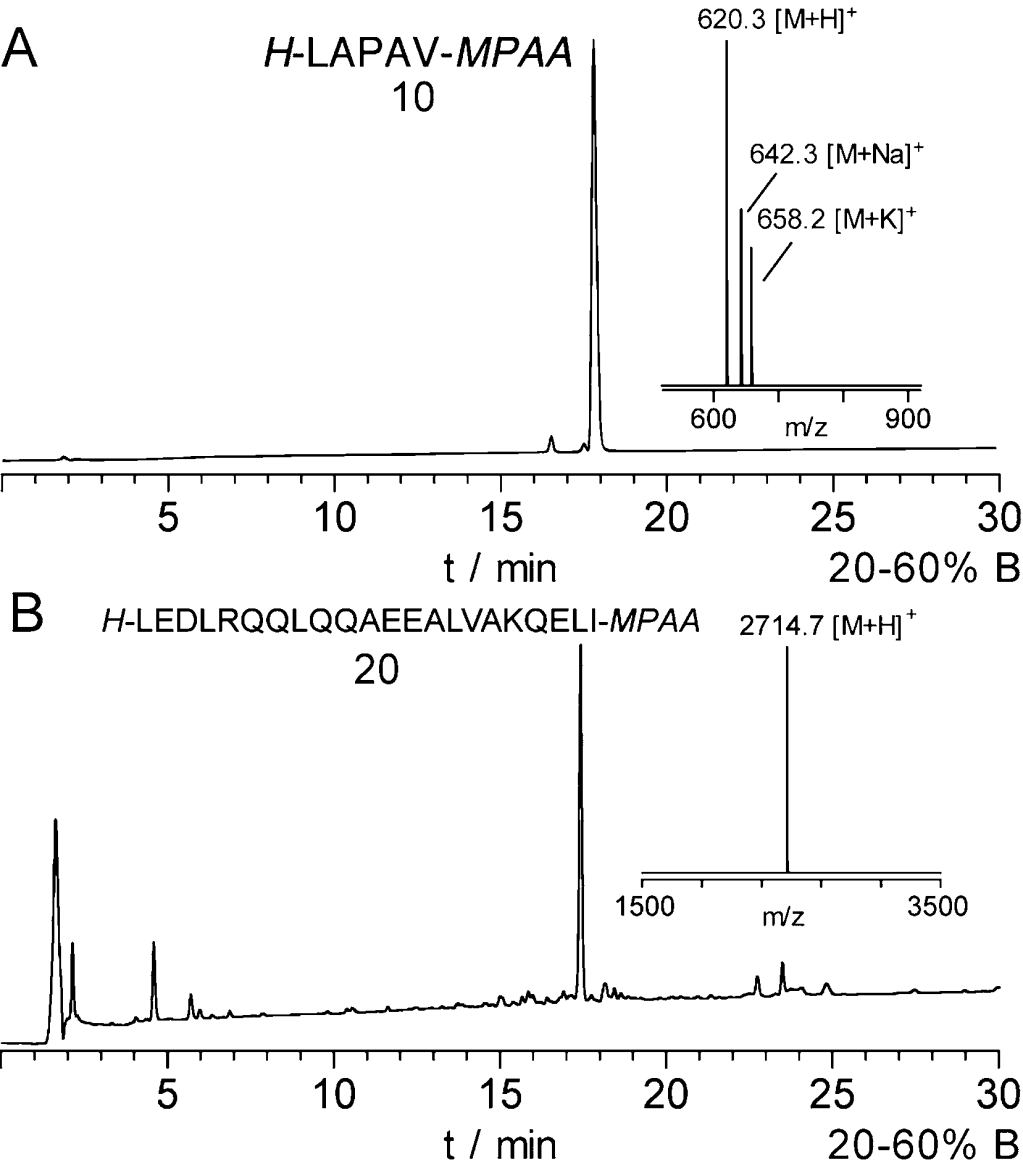
Crude HPLC traces and MALDI‐TOF MS of MPAA thioesters synthesized using Boc protocols on Merrifield hydroxymethyl resin. The peptides where cleaved with TFA/TMSBr/thioanisole/EDT (1:0.05:0.05:0.025) for 1.5 h at room temperature and precipitated from Et_2_O. A) LAPAV‐MPAA and B) LEDLRQQLQQAEEALVAKQELI‐MPAA.

One of the advantages of this method is its greater compatibility with post‐translational modifications compared to conventional Boc SPPS. We illustrated the scope of the technique by the preparation of phosphorylated CHK2 protein. CHK2 is a serine/threonine kinase which upon activation by phosphorylation on Thr‐68 plays a central role in DNA damage response.^27^ Furthermore, it is involved in cell cycle checkpoint activation, apoptosis, viral infectivity and other pathways.^28^ The C‐terminal portion bearing an N‐terminal cysteine was prepared by expression. The N‐terminal peptide has been previously synthesized by Fmoc SPPS using the sulfonamide safety catch method.^29^ Phosphorylated threonine was added to the synthesis as the building block Boc‐Thr‐(PO_3_Me_2_)‐OH. A 2.5‐fold excess of phosphorylated MPAA thioester **15** was ligated with truncated CHK2 protein (100 μm concentration) in phosphate buffer (200 mm; pH 7.0). The ligation was monitored by gel electrophoresis and was complete after 60 min and the further addition of another 2.5‐fold of peptide **15** (Figure 3 B). Phosphorylated CHK2 protein dimerized confirming successful ligation (Figure 3 D).^29^

**Figure 3:**
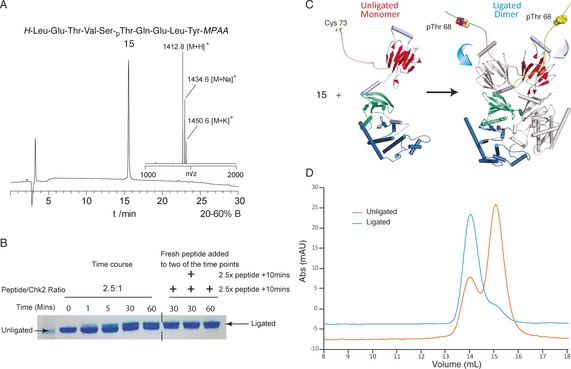
Synthesis and application of an MPAA thioester phosphopeptide. A) Analytical HPLC (20–60 % B=CH_3_CN/H_2_O/TFA (90:10:0.1) in 30 min) and MALDI‐TOF MS of the purified phosphopeptide **15**. B) Time course of the ligation of **15** with CHK2(73‐538)S73C. The lower band is CHK2(73‐538)S73C and the upper band is the ligated protein. After 60 min, approximately 50 % ligation was observed. Additional peptide was added at the 30 min and 60 minute time points, and the ligation proceeded to >90 % conversion. The ligation product contains the residues 63–538 with a phosphorylated Thr‐68 and a Ser‐73 to Cys mutation. C) Ligation of phosphopeptide **15** to CHK2(73‐538)S73C and dimerization of phosphoprotein. D) Gel‐filtration profiles of the purified unligated CHK2(73‐538)S73C and ligated pT68CHK2. Indicating dimerization for ligated material, small amount of dimerization was also present for the unligated protein.

Another application of peptide thioesters is for the synthesis of cyclic peptides. With many leads being identified from the screening of natural products and selection technologies^30^ chemistry remains the bottleneck to their large‐scale preparation. Peptide thioesters are particularly adept at cyclization by NCL.2a We demonstrated this with the synthesis of **18** (Table 1) which contained a good range of residues including tryptophan. Side‐chain unprotected tryptophan was used in the synthesis, a standard procedure for peptide thioester synthesis by Boc SPPS. The peptide was dissolved in phosphate buffer containing MPAA. Cyclization monitored by analytical HPLC was complete after 15 min (Figure S22, SI).

Peptide thioesters have been successfully cyclized in the absence of N‐terminal cysteine. Recently, peptide thiophenyl esters were shown to cyclize.2c Independently, the Houghten group cyclized peptide MPAL thioesters.2b We decided to repeat the conditions of Houghten and co‐workers but using MPAA thioesters. The target we chose was the KRAS inhibitor cyclorasin 9A5.^31^ The analytical HPLC of the crude linear peptide **17** was very clean (Figure 4 A) considering it possessed four arginines and a tryptophan.

**Figure 4:**
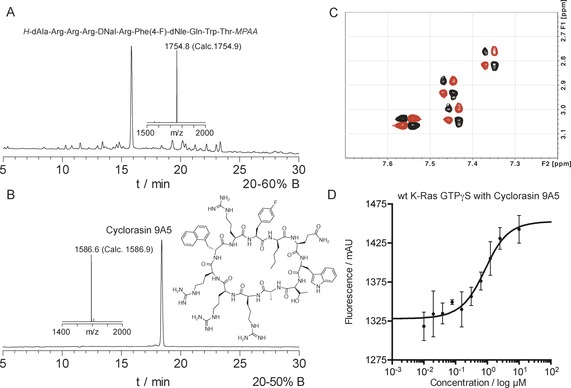
Synthesis, cyclization and characterization of cyclorasin 9A5. A) Analytical HPLC of crude linear cyclorasin 9A5 precursor synthesized as an activated MPAA thioester. Insert shows MALDI‐TOF MS of the purified product. B) Analytical HPLC and MALDI‐TOF MS of purified cyclized cyclorasin 9A5, **24**. The crude linear peptide (1 mm) was cyclized by adding to a mixture of MeCN:H_2_O (7:1) and imidazole. The reaction was monitored to completion by analytical HPLC, cyclorasin 9A5 was purified by semi‐preparative HPLC. C) COSY NMR spectrum of cyclorasin 9A5. COSY showing four distinct Arg H(δ)–H(ϵ) correlations, indicating head‐to‐tail topology. D) Binding study of cyclorasin 9A5 with wild‐type KRAS protein. Microscale thermophoresis studies were performed in triplicate showing a *K*_d_ of 587 nm (see Figure S24 in the SI for biophysical characterization).

Cyclization in acetonitrile in the presence of an aqueous imidazole solution gave good results (Figure S23, SI). The head‐to‐tail connectivity of cyclorasin 9A5 was confirmed by NMR spectroscopy (Figure 4 C). The binding was characterized by microscale thermophoresis studies (Figure 4 D). Labeled KRAS‐GTPγS interacts with cyclorasin 9A5 with an affinity of 0.6 μm similar to the values measured previously with FITC‐labeled peptide^31^ and the dissociation constant between cyclorasin and wild‐type KRAS was measured (Figure 4 D and SI).

In summary we have reported a direct Boc SPPS approach for the synthesis of peptide thioesters with a TFA/TMSBr cleavage replacing HF treatment. This is a simple, practical method with a gentler cleavage step, compatible with many post‐translational modifications. The avoidance of HF makes Boc SPPS much more accessible. The in situ neutralization cycles, effective for overcoming difficult sequences provide peptides of high purity, difficult to achieve by Fmoc SPPS without the often problematic use of backbone protection.^6^

## Supporting information

As a service to our authors and readers, this journal provides supporting information supplied by the authors. Such materials are peer reviewed and may be re‐organized for online delivery, but are not copy‐edited or typeset. Technical support issues arising from supporting information (other than missing files) should be addressed to the authors.

Supplementary
