# Supplementary material for: HF‐Free Boc Synthesis of Peptide Thioesters for Ligation and Cyclization
Source: Angew Chem Int Ed Engl. 2016 Oct 6;55(42):13174–9. doi: 10.1002/anie.201607657 (PMC5113665; doi:10.1002/anie.201607657)
Supplement: Supplementary file 1 — Supplementary [file ANIE-55-13174-s001.pdf]

Angewandte Chemie
Eine Zeitschrift der Gesellschaft Deutscher Chemiker

Supporting Information

HF-Free Boc Synthesis of Peptide Thioesters for Ligation and Cyclization

*Richard Raz, Fabienne Burlina, Mohamed Ismail, Julian Downward, Jiejin Li, Stephen J. Smerdon, Martin Quibell, Peter D. White, and John Offer**

anie_201607657_sm_miscellaneous_information.pdf

Supporting Information

Table of Contents

(I) General Methods	3
(II) TFA stability tests	6
(III) Peptide synthesis	7
(IV) Peptide Cyclisations	22
(V) CHK2 phosphopeptide ligation	25
(VI) Biological analysis of Cyclorasin 9A5	26
(VII) References	28

I. General Methods

Abbreviations:

CHCA: α-cyano-4-hydroxycinnamic acid. EtOAc: ethyl acetate. DIC: 1,3-diisopropylcarbodiimide. DIEA: N,N-diisopropylethylamine. DMF: N,N-dimethylformamide. DMAP: 4-(Dimethylamino)pyridine. EDTA: ethylenediamine tetraacetic acid. HCTU: (2-(6-Chloro-1H-benzotriazole-1-yl)-1,1,3,3-tetramethylaminium hexafluorophosphate). HOBt: 1-hydroxybenzotriazole. EDT: Ethanedithiol. TES: Triethylsilane MALDI-TOF MS: matrix assisted laser desorption ionization time-of-flight mass spectrometry. MPAA: 4-mercaptophenylacetic acid. MPAL: mercaptopropionic acid leucine. rt: room temperature. TCEP: tris(2-carboxyethyl)phosphine. TFA: trifluoroacetic acid. TMSBr: Trimethylsilyl bromide. TLC: thin-layer chromatography.

Peptide Synthesis General Information:

Boc-amino acids were purchased from Merck Novabiochem unless otherwise stated, Boc-Arg(Mts)-OH was purchased from CS Bio, Menlo Park (CAS no. 102185-38-6). Boc-Gln(Xan)-OH was purchased from Anaspec (CAS no. 55260-24-7). Boc-Thr(PO_3Me_2)-OH was purchased from Sigma (CAS no. 162554-18-9). 3-tritylmercaptopropionic acid was purchased from Bachem (CAS no. 27144-18-9). TMSBr was purchased from Sigma (CAS no. 2857-97-8). Peptides were prepared by standard manual Boc-SPPS methods using a glass filter frit. Following peptide-resin cleavage, the TFA/TMSBr-cleavage cocktail was filtered, filtrate sparged (nitrogen) and peptides were precipitated with Et_2O (Na-dried, 4 °C), dried *in vacuo* then re-dissolved in water and acetonitrile mixtures and freeze-dried.

Preparative reverse-phase HPLC was performed using a Waters 600 System and Waters Delta 600 pump with a Waters 2489 uv/vis detector. Peptides were purified by semi-preparative HPLC on an RP-C18 column (22 x 250 mm, Vydac) using linear gradients of CH_3CN in 0.1 % TFA/H_2O with a flow rate of 15 mL min^{-1}. HPLC gradients were prepared using solvent A (0.1% TFA/H_2O) and solvent B (90 % CH_3CN/H_2O with 0.1 % TFA).

Analytical reverse-phase HPLC was performed either on an Agilent Technologies 1200 Series System with a uv vis detector or a Hitachi Elite Lachrom system with an L-2455 diode array detector. Peptides were analysed on a Phenomenex Gemini 3 μm analytical column (150 x 4.6 mm), using linear gradients of CH_3CN in 0.1 % TFA/H_2O with a flow rate of 1 mL min^{-1}. Detection was performed at 214 nm.

Peptides were characterized by MALDI-TOF MS on a BRUKER microflex (ion positive linear and reflectron mode) using CHCA matrix (10 mg mL^{-1} in CH_3CN/H_2O/TFA, 50:50:0.1).

Table S1. Protecting group strategy for alternative Boc-SPPS adapted for TFA/TFMSBr[S1]

Amino acid	Side-chain Protection HF cleavage[a]	Side-chain Protection TFA/TMSBr	Deprotection time- using TFA/TMSBr[b]
Arg	Tos	Mts	1-3 h
Asp	cHx	Bzl[c]	1 h
Glu	cHx, Bzl	Bzl[c]	1 h
Trp	Formyl	None	~
His	Dnp	Dnp	not removed
Cys	Meb	Mob	< 0.5 h
Ser, Thr	Bzl	Bzl	1 h
Tyr	BrZ	BrZ	1 h
Lys	ClZ	ClZ	1 h

[a] As used for contemporary in situ neutralisation Boc SPPS [b] Estimated time for complete removal [c] Bzl was the original protection for Asp and Glu used in early Boc SPPS replaced by OcHx with introduction of HF.

Preparation of the mercaptopropionic acid leucine (*MPAL*) resin:

Resin loading was adapted from Hackeng et al.[S2] and performed on small batches typically: hydroxymethyl resin (0.250 g, 0.316 mmol, Merck Chemicals) was swollen in dry CH_2Cl_2 for at least 2 h and derivatised as follows. DIC (0.2 g, 1.58 mmol, 5 eq.) in CH_2Cl_2 (2 mL) was added to a suspension of Boc-Leu-OH (0.80 g, 3.15 mmol, 10 eq.) in CH_2Cl_2 (2 mL) over 10 min at 0 °C followed by addition of DMAP (0.038 g, 0.315 mmol, 1 eq.). The resulting suspension was warmed to room temperature and stirred for 45 min to allow complete formation of the symmetric anhydride which was then added to the swollen hydroxymethyl Merrifield resin. The resin was left overnight and thoroughly washed with CH_2Cl_2 and DMF. The Boc-group was removed using neat TFA (2 x 1 min) and washed with DMF. DIEA was added until fuming ceased to neutralize any remaining TFA. 3-Tritylmercaptopropionic acid (0.44 g, 1.26 mmol, 4 eq.) was activated with HCTU (0.52 g, 1.26 mmol, 4 eq.) and DIEA (0.12 g, 0.95 mmol, 3 eq.) for 3 min and added to the resin. For peptide elongation of the *MPAL* resin, the trityl group was removed with treatment with a TFA mixture (2.5 % TES, 2.5 % H_2O in TFA, 3 x 2 min until all yellow color had disappeared). The resulting *MPAL* resin was used for polypeptide chain assembly by the implementation of *in-situ* neutralization SPPS protocols.

Preparation of the trityl-mercaptophenylacetic acid (Trt-MPAA):

The title compound was adapted from Dang et al.[S3] briefly, trityl chloride (1.0 g, 3.59 mmol) was added to a suspension of 4-mercaptophenylacetic acid (0.559 g, 3.32 mmol) in CH_2Cl_2 (10 mL), stirred for 3 h, and the reaction monitored by TLC to completion. The reaction was quenched with the addition of H_2O (10 mL) and extracted with EtOAc (1 x 40 mL). The organic phase was washed with brine (1 x 30 mL), dried (Na_2SO_4) and evaporated. Silica Chromatography (3:1 Petroleum ether: EtOAc on silica gel) yielded 4-(tritylmercapto)phenylacetic acid (Trt-MPAA) (1.05 g, 77 %) as a white crystalline solid.

^{1}H-NMR: (400 MHz, $CDCl_3$) δ [ppm]: 7.41-7.37 (m, 6H), 7.28-7.15 (m, 9H), 6.91-6.89 (m, 4H), 3.49 (s, 2H).

Preparation of the Trt-MPAA-resin general method:

Method A: Resin loading was adapted from Topping et al., and performed typically on 0.1 mmol batches of resin.[S4] Merrifield hydroxymethyl resin (0.5-1.0 mmol/g, 1 g, Sigma Aldrich) was swollen in dry CH_2Cl_2 for at least 2 h and drained. The resin was then suspended in PBr_3 (1.0 M in CH_2Cl_2; 5 mL) and shaken (1 h, rt), then washed with CH_2Cl_2 (5 x 5 mL) and DMF (5 x 5 mL). Addition of the MPAA building block was accomplished with Trt-MPAA (0.451 g, 1.1 mmol, 1.1 eq.), DIEA (0.278 g, 0.374 mL, 2.2 mmol, 2.2 eq.) in dry DMF and shaken (18 h). The resin was washed with DMF, CH_2Cl_2 and dried in vacuo to give Trt-MPAA loaded resin (1.564 g).

Method B: In a small glass vial, hydroxymethyl resin (1.26 mmol/g, 0.158 g, 0.2 mmol, Merck Chemicals) was swollen in CH_2Cl_2 for 3 h. In a separate glass vial, Trt-MPAA (0.246 g, 0.6 mmol, 3 eq.), DIC (0.075 g, 0.6 mmol, 3 eq.), and DMAP (0.024 g, 0.2 mmol, 1 eq.) was dissolved in CH_2Cl_2 (2 mL) and added to the resin. The resin was shaken (1 h, rt) drained and washed with DMF (5 x 30 s). To cap any remaining free hydroxyl sites, Ac_2O (1 mL) and DIEA (2 mL) in DMF (7 mL) were added to the resin and shaken (30 min). The resin was drained and washed with DMF.

***In-situ* neutralisation SPPS protocols:**

Boc-SPPS in situ neutralisation protocols were adapted from Schnolzer et al.[S5] The Boc-amino acid (1.26 mmol, 4 eq.) and HCTU (1.197 mmol, 3.8 eq.) were dissolved in DMF (2.5 mL). DIEA (1.89 mmol, 6 eq.) was added, the coupling mixture was shaken for 3 min and added to a reaction vessel containing the *MPAL* resin. The reaction was left for 30 min followed by a DMF flow-wash (2 x 30 s).

Coupling of the remaining amino acids was accomplished as follows: After removal of the Boc-group using neat TFA (2 x 1 min), the resin was flow-washed vigorously with DMF (3 x 10 mL) for no more than 30 s under concurrent draining via vacuum. The next amino acid was then activated as previously described and added to the resin to couple for 30 min.

TFA/TMSBr Cleavage:

After coupling of the last amino acid, the resin was washed with DMF and CH_2Cl_2 and dried in vacuo. Cleavage from the resin and total deprotection was accomplished

with TFA/TMSBr/Thioanisole/EDT (1/0.05/0.05/0.025) (typically 5 mL per 0.1 g of peptide-resin). The TMSBr bottle was pressurised with Argon, and TMSBr was transferred via syringe and canula to the reagent vessel. The volatiles were sparged under a stream of N_2, the peptide was precipitated from Et_2O (Na-dried, 4 °C). HPLC Purification of the crude peptide was performed on a C18 column (see General Methods) the fractions were collected and lyophilized to give the purified peptide as a white powder.

II: TFA stability tests

Fmoc-Gly-resin and Fmoc-Gly-*MPAL*-resin were prepared as previously described, using PBr_3 for the method of attachment to the solid support. Upon completion of the synthesis, the resin was washed with DMF (2 x 30 s continuous flow), CH_2Cl_2 (2 x 30 s. continuous flow), and dried overnight in vacuo. The dried resins (6.52 mg for Fmoc-Gly-resin and 6.97 mg for Fmoc-Gly-*MPAL*-resin) were treated with TFA (10 mL) for 8 h. Time points were taken at 10 min, 30 min, 1 h, 2 h, 3 h, 4 h, 5 h, 6 h, 7 h, 8 h, and the absorbance measured at 301 nm with a Jasco UV-Vis Spectrometer employing glass suprasil cuvettes with a path length of 10 mm. The data was fitted with a non-linear curve using Graph Pad Prism 6.0 to determine half-life stabilities of the measured resins.

III: Peptide Synthesis

Non-thioester Syntheses:

***H*-PGVGPGVGV-*OH* (Elastin) (1)**. The peptide was synthesized on a Merrifield hydroxymethyl resin (0.2 g, 0.2 mmol) using standard *in situ* neutralisation protocols for Boc SPPS. 0.382 g of resin was obtained upon completion of the synthesis (98 % yield based on expected dry weight). After TFA/TMSBr cleavage and precipitation from Et_2O (Na-dried, 4 °C) from a portion of 170 mg, the title compound was obtained as a white powder (0.080 g, 81 %) (purity > 95 %) Analytical HPLC of *H*-PGVGPGVGV-*OH* **1**: R_t 18.8 min (5-50 % B over 30 min, λ = 214 nm); MALDI-TOF-MS: m/z = 837.6 $[M+H]^+$ (First isotope), calc.: 837.5.

***H*-GCCSDPRCRYRCR-*OH* (α-Conotoxin RgIA) (2)**. The peptide was synthesised on a Merrifield hydroxymethyl resin (0.2 g, 0.1 mmol) using standard *in situ* neutralisation protocols for Boc SPPS. 0.406 g of resin was obtained upon completion of the synthesis (77 % yield based on expected dry weight). After TFA/TMSBr cleavage of 106 mg peptidyl-resin preparative HPLC yielded pure **2** (23 mg, 21 %)

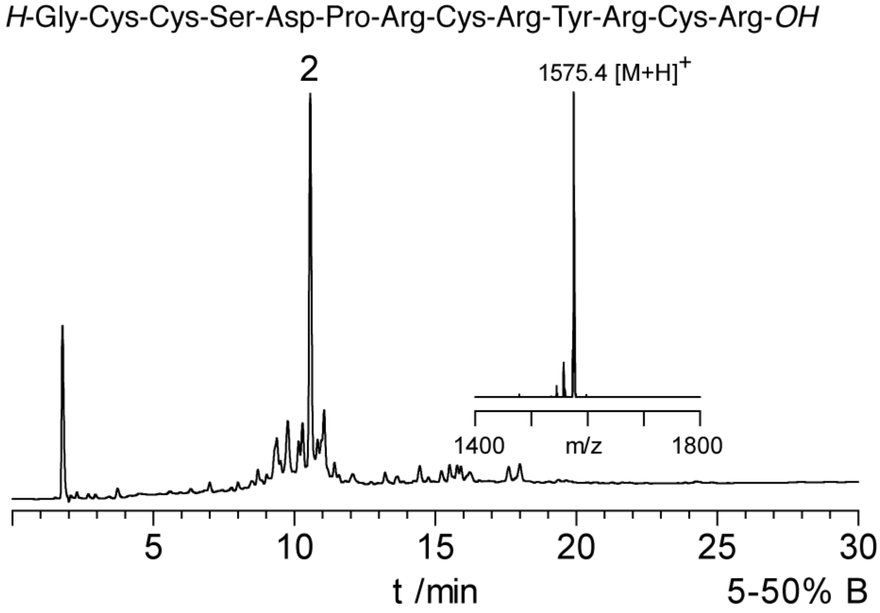

Figure S1. Analytical HPLC of crude α-Conotoxin RgIA **2**: R_t 10.5 min (5-50 % B over 30 min, λ = 214 nm); MALDI-TOF-MS: m/z = 1575.4 $[M+H]^+$ (First isotope), calc.: 1574.7.

MPAL **thioester syntheses**:

***H*-LAPAV-*MPAL* (3)**. Starting with Merrifield hydroxymethyl resin (0.25 g, 0.315 mmol), peptide **3** was prepared using the *MPAL* method described above and synthesised using in situ neutralisation protocols for Boc SPPS. 0.448 g of resin was obtained upon completion of the synthesis (92 % yield based on expected dry weight from a 0.315 mmol scale). The peptide-resin was cleaved using TFA/TMSBr for 1 h as described above. Preparative HPLC (15-40 % B in 30 min), yielded the title compound *H*-LAPAV-*MPAL* **3** as a white powder (0.114 g, 54 %) (purity > 98 %) (Figure 1D).

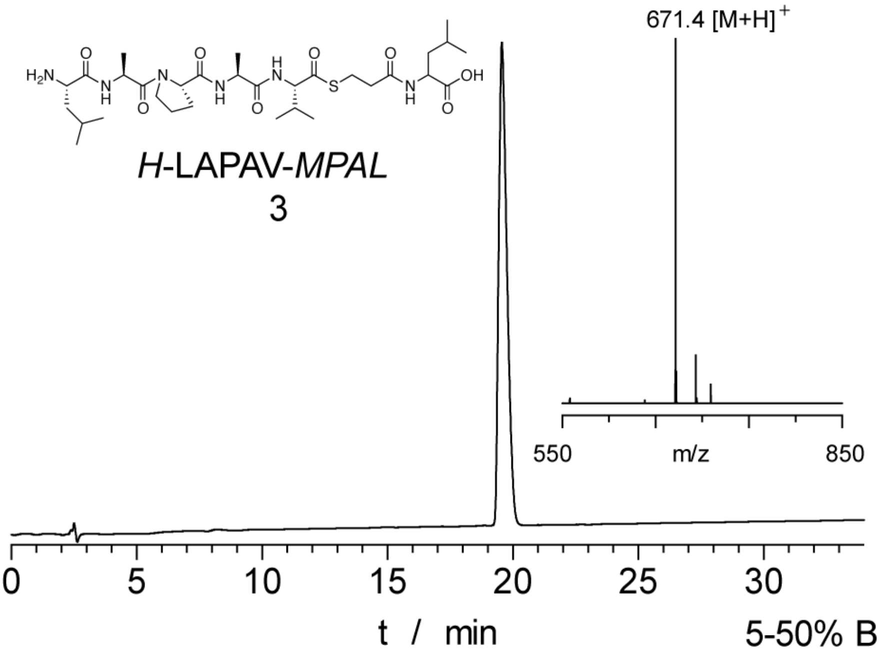

Figure S2. Analytical HPLC of pure *H*-LAPAV-*MPAL* **3**: R_t 19.6 min (5-50% B over 30 min, λ = 214 nm); MALDI-TOF-MS: m/z = 671.5 $[M+H]^+$ (First isotope), calc.: 671.4.

***H*-LAPAA-*MPAL* (4)**. Starting with Merrifield hydroxymethyl resin (0.25 g, 0.315 mmol), peptide **4** was prepared using the *MPAL* method described above and synthesised using in situ neutralisation protocols for Boc SPPS. 0.433 g of resin was obtained upon completion of the synthesis (90 % yield based on expected dry weight from a 0.315 mmol scale). The peptide-resin was cleaved using TFA/TMSBr for 1 h. Preparative HPLC (15-40 % B in 30 min), yielded the title compound *H*-LAPAA-*MPAL* **4** as a white powder (0.101 g, 50 %) (purity > 98 %).

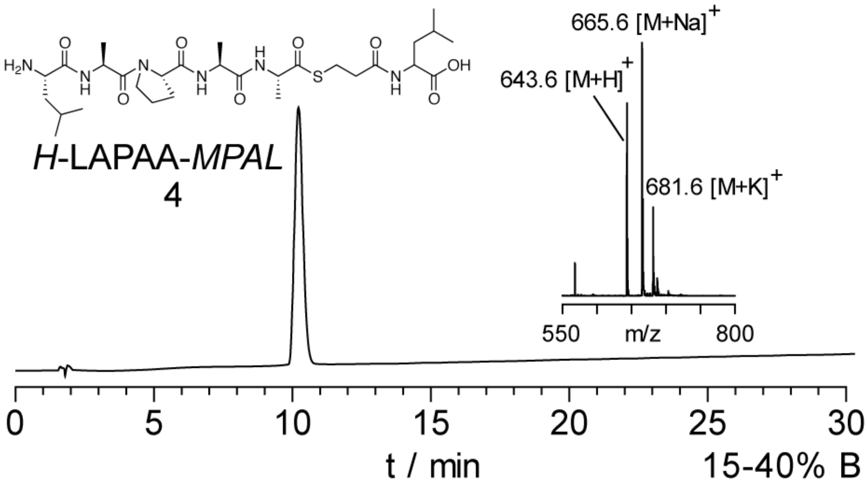

Figure S3. Analytical HPLC of pure *H*-LAPAA-*MPAL* **4**: R_t 10.3 min (15-40 % B over 30 min, λ = 214 nm); MALDI-TOF-MS: m/z = 643.6 $[M+H]^+$ (First isotope), calc.: 643.4.

***H*-LAPAG-*MPAL* (5)**. Starting with Merrifield hydroxymethyl resin (1.26 g, 1.0 mmol), peptide **5** was prepared using the *MPAL* method described above and synthesised using in situ neutralisation protocols for Boc SPPS. 1.53 g of resin was obtained upon completion of the synthesis (86 % yield based on expected dry weight from a

1.0 mmol scale). The peptide-resin was cleaved using TFA/TMSBr for 1 h. Preparative HPLC (15-40 % B in 30 min), yielded the title compound *H*-LAPAG-*MPAL* **5** as a white powder (0.201 g, 22 %) (purity > 98 %).

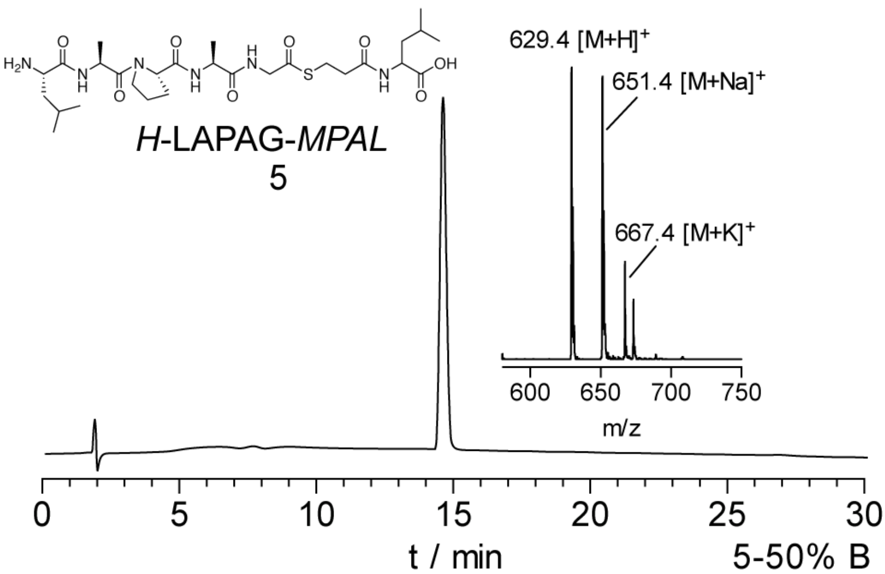

Figure S4. Analytical HPLC of pure *H*-LAPAG-*MPAL* **5**: R_t 14.8 min (5-50% B over 30 min, λ = 214 nm); MALDI-TOF-MS: m/z = 629.4 $[M+H]^+$ (First isotope), calc.: 629.3.

***H*-LYRAF-*MPAL* (6)**. Starting with Merrifield hydroxymethyl resin (0.25 g, 0.315 mmol), peptide **6** was prepared using the *MPAL* method described above and synthesised using in situ neutralisation protocols for Boc SPPS. 0.554 g of resin was obtained upon completion of the synthesis (81 % yield based on expected dry weight from a 0.315 mmol scale). The peptide-resin was cleaved using TFA/TMSBr for 2 h. Preparative HPLC (15-40 % B in 30 min), yielded the title compound *H*-LYRAF-*MPAL* **6** as a white powder (0.116 g, 34 %) (purity >98 %).

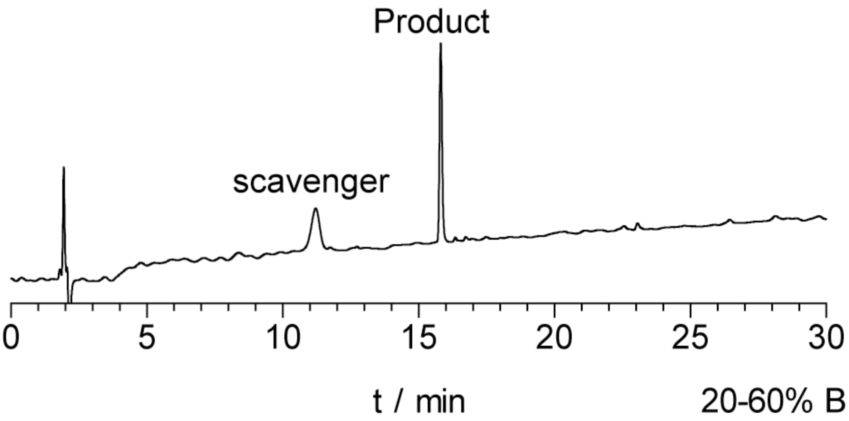

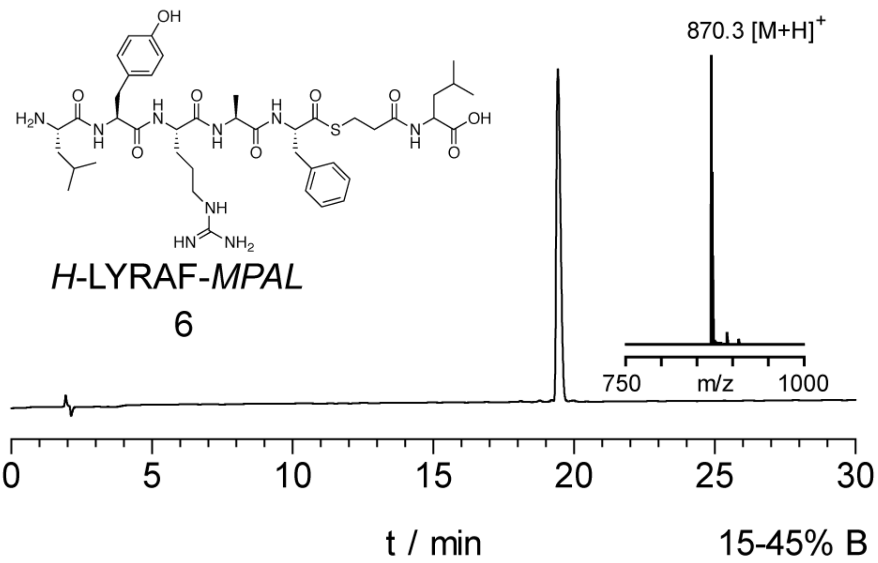

Figure S5. Above: Analytical HPLC of crude peptide *H*-LYRAF-*MPAL* **6:** R_t 15.9 min (20-60 % B over 30 min, λ = 214 nm). Below: Analytical HPLC of pure peptide *H*-LYRAF-*MPAL*: R_t 19.4 min (15-45 % B MALDI-TOF-MS: m/z = 870.3 $[M+H]^+$ (First isotope), calc.: 870.5.

MPAA thioester syntheses:

***H*-LAPAG-*MPAA* (7)**. Starting with Merrifield hydroxymethyl resin (0.17g, 0.2 mmol) prepared using Method A, peptide **7** was synthesised using in situ neutralisation protocols for Boc SPPS. 0.274 g of dried resin was obtained upon completion of the synthesis (68 % yield based on expected dry weight from a 0.2 mmol scale). The peptide-resin was cleaved using TFA/TMSBr for 1 h. Preparative HPLC (15-40 % B in 30 min), yielded the title compound *H*-LAPAG-*MPAA* **7** as a white powder (0.024 g, 16 %) (purity > 98 %).

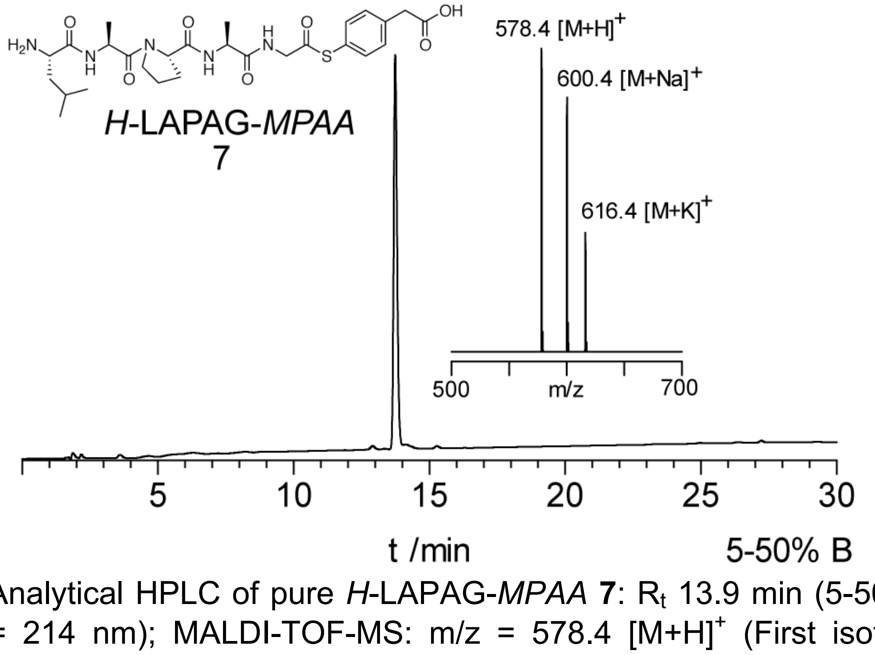

Figure S6. Analytical HPLC of pure *H*-LAPAG-*MPAA* **7**: R_t 13.9 min (5-50 % B over 30 min, λ = 214 nm); MALDI-TOF-MS: m/z = 578.4 $[M+H]^+$ (First isotope) Calc: 578.3.

***H*-LAPAA-*MPAA* (8).** Starting with Merrifield hydroxymethyl resin (0.1 g, 0.08 mmol) prepared using Method A, peptide **8** was synthesised using in situ neutralisation protocols for Boc SPPS. 0.11 g of dried resin was obtained upon completion of the synthesis (84 % yield based on expected dry weight from a 0.08 mmol scale). The peptide-resin was cleaved using TFA/TMSBr for 1 h. Preparative HPLC (15-40 % B in 30 min), yielded the title compound *H*-LAPAA-*MPAA* **8** as a white powder (0.015 g, 31 %) (purity > 98 %).

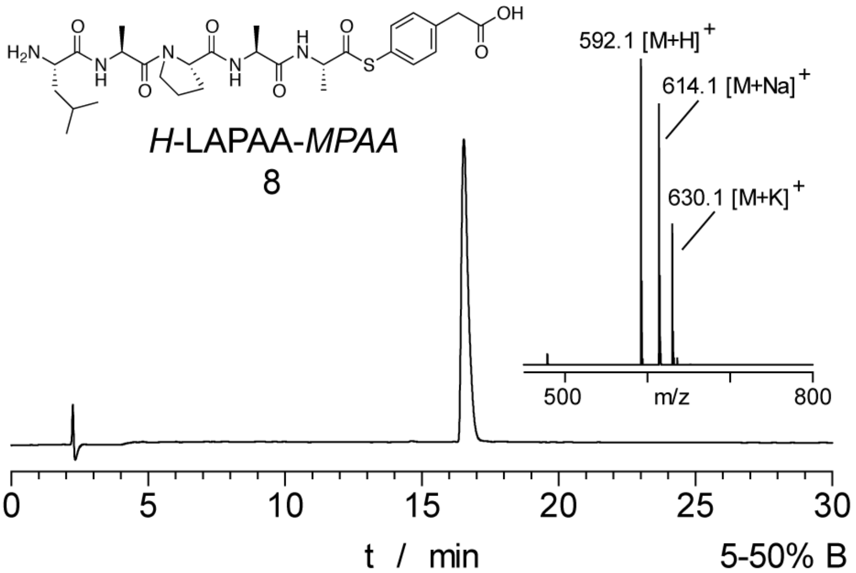

Figure S7. Analytical HPLC of pure *H*-LAPAA-*MPAA* **8**: R_t 13.9 min (5-50 % B over 30 min, λ = 214 nm); MALDI-TOF-MS: m/z = 592.1 $[M+H]^+$ (First isotope), calc.: 592.3.

***H*-LAPAQ-*MPAA* (9).** Starting with Merrifield hydroxymethyl resin (0.13 g, 0.1 mmol) prepared using Method A, peptide **9** was synthesised using in situ neutralisation protocols for Boc SPPS. 0.143 g of dried resin was obtained upon completion of the synthesis (83 % yield based on expected dry weight from a 0.1 mmol scale). The peptide-resin was cleaved using TFA/TMSBr for 1 h. Preparative HPLC (20-50 % B in 30 min), yielded the title compound *H*-LAPAQ-*MPAA* **9** as a white powder (0.020 g 26 %) (purity >98 %).

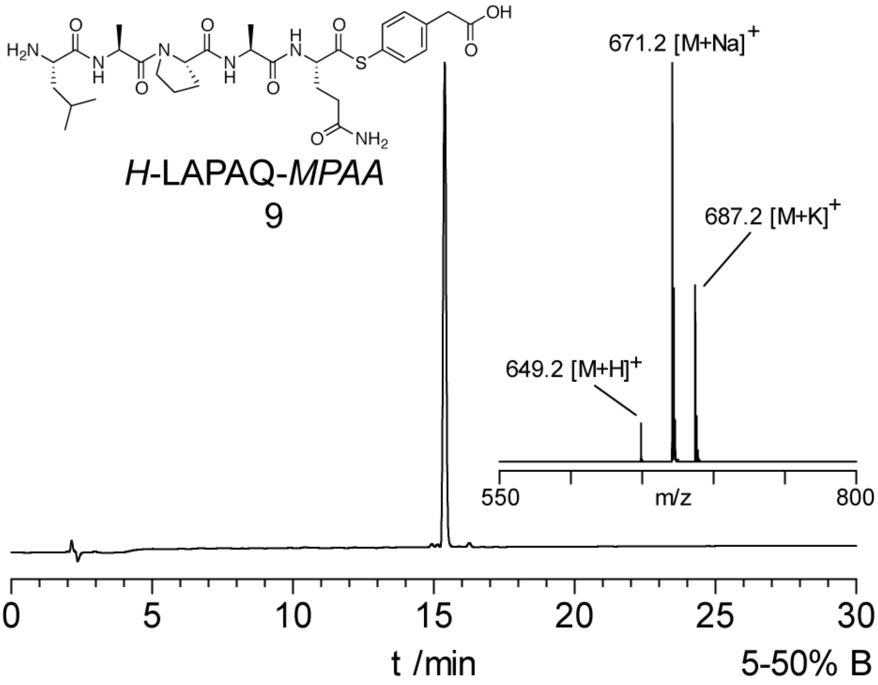

Figure S8. Analytical HPLC of pure *H*-LAPAQ-*MPAA* **9**: R_t 13.9 min (5-50 % B over 30 min, λ = 214 nm); MALDI-TOF-MS: m/z = 649.2 $[M+H]^+$ (First isotope), calc.: 649.3.

***H*-LAPAV-*MPAA* (10).** Starting with Merrifield hydroxymethyl resin (0.11 g, 0.1 mmol) prepared using Method A, peptide **10** was synthesised using in situ neutralisation protocols for Boc SPPS. 0.164 g of dried resin was obtained upon completion of the synthesis (89 % yield based on expected dry weight from a 0.1 mmol scale). The peptide-resin was cleaved using TFA/TMSBr for 1 h. Preparative HPLC (15-40 % B in 30 min), yielded the title compound *H*-LAPAV-*MPAA* **10** as a white powder (0.030 g, 36 %) (purity > 98 %).

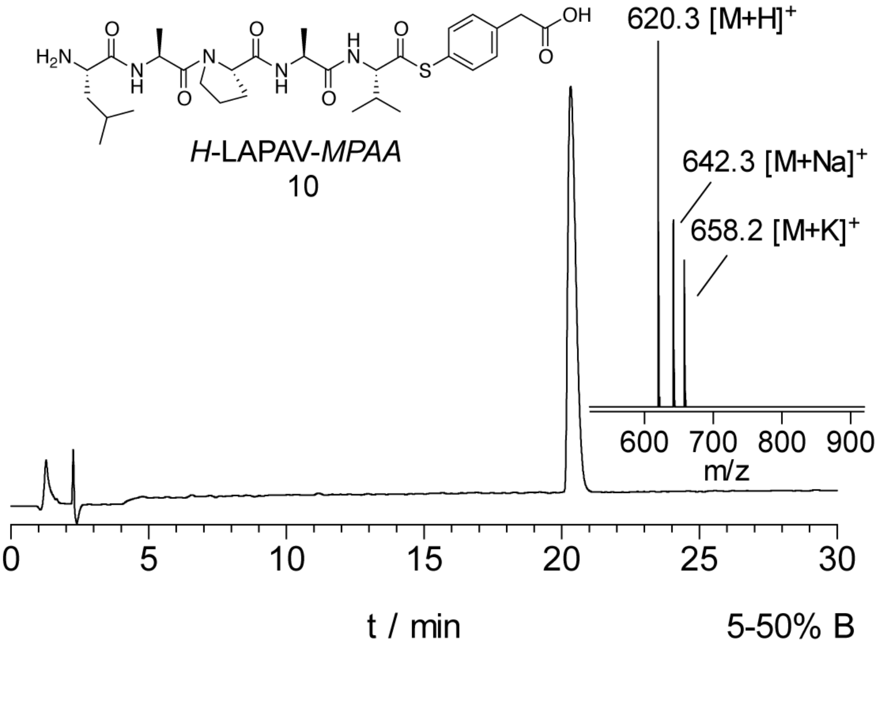

Figure S9. Analytical HPLC of pure *H*-LAPAV-*MPAA* **10**: R_t 20.5 min (5-50% B over 30 min, λ = 214 nm); MALDI-TOF-MS: m/z = 620.3 $[M+H]^+$ (First isotope), calc.: 620.3.

***H*-LAPAT-*MPAA* (11).** Starting with Merrifield hydroxymethyl resin (0.1 g, 0.08 mmol) prepared using Method A, peptide **11** was synthesised using in situ neutralisation protocols for Boc SPPS. 0.114 g of dried resin was obtained upon completion of the synthesis (86 % yield based on expected dry weight from a 0.1 mmol scale 0.08 mmol scale). The peptide-resin was cleaved using TFA/TMSBr for 1 h. Preparative HPLC (20-50 % B in 30 min), yielded the title compound *H*-LAPAT-*MPAA* **11** as a white powder (0.027 g, 36 %) (purity > 98 %).

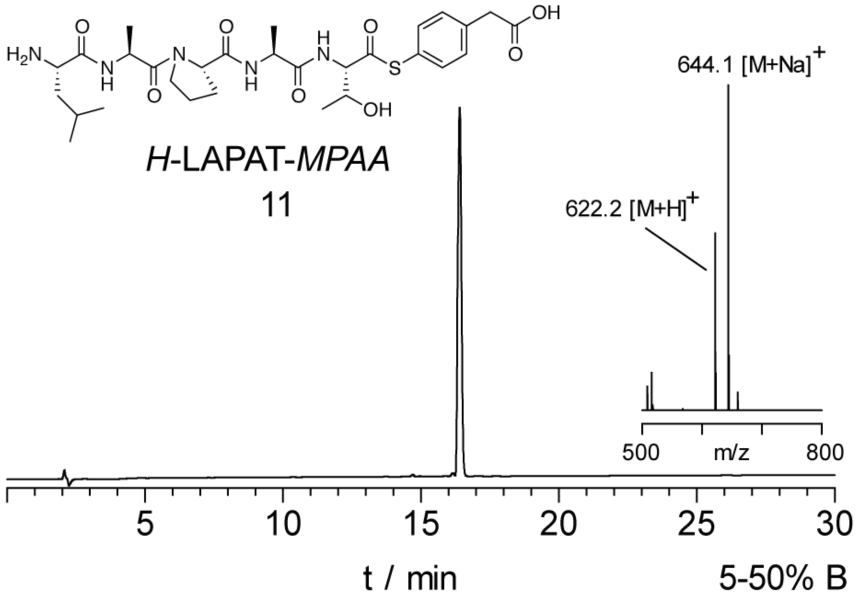

Figure S10. Analytical HPLC of pure *H*-LAPAT-*MPAA* **11**: R_t 16.4 min (5-50 % B over 30 min, λ = 214 nm); MALDI-TOF-MS: m/z = 622.2 $[M+H]^+$, (First isotope), calc.: 622.3.

***H*-LAPAW-*MPAA* (12).** Starting with Merrifield hydroxy methylresin 0.13 g (0.1 mmol) prepared using Method A, peptide **12** was synthesised using in situ neutralisation protocols for Boc SPPS. 0.149 g of dried resin was obtained from the synthesis (83 % yield based on expected dry weight from a 0.1 mmol scale). The peptide-resin was cleaved using TFA/TMSBr for 1 h. Preparative HPLC (25-55 % B in 30 min), yielded the title compound *H*-LAPAW-*MPAA* **12** as a white powder (0.022 g, 26 %) (purity > 98 %).

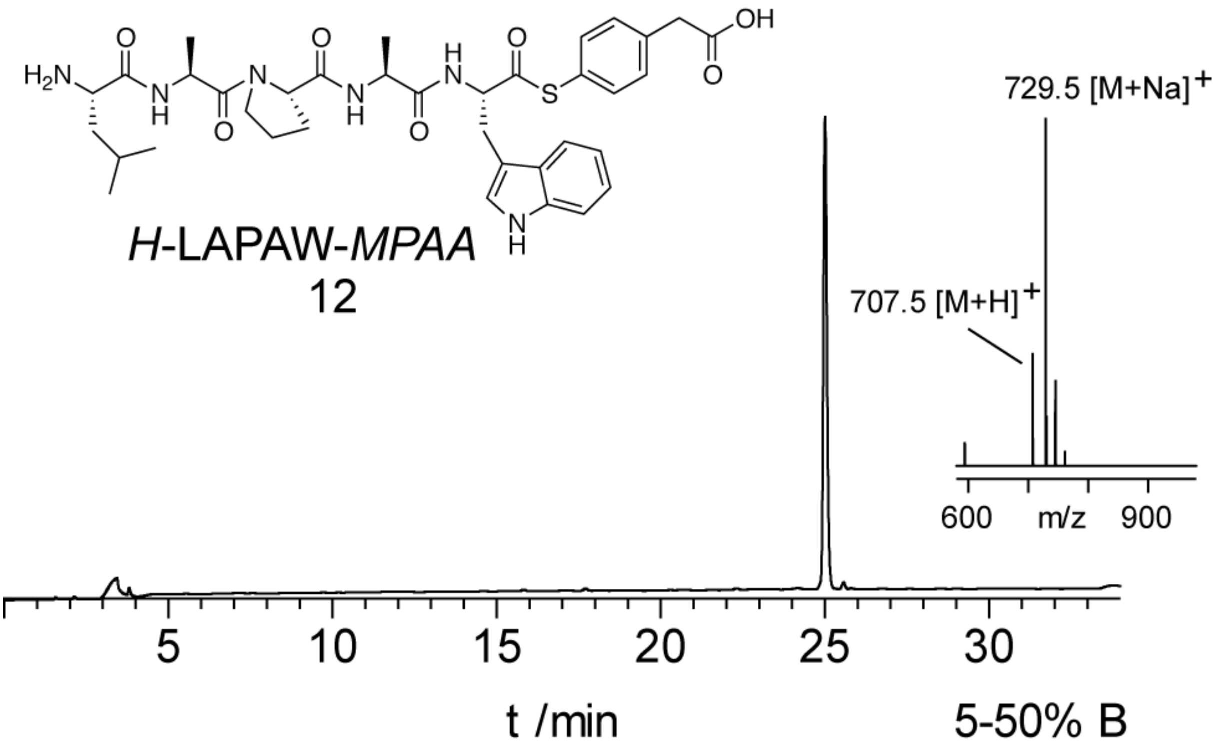

Figure S11. Analytical HPLC of pure *H*-LAPAW-*MPAA* **12**: R_t 25.0 min (5-50 % B over 30 min, λ = 214 nm); MALDI-TOF-MS: m/z = 707.5 $[M+H]^+$, 729.5 $[M+Na]^+$ (First isotope), calc.: 707.3, 729.3.

***H*-LYRAI-*MPAA* (13).** Starting with Merrifield hydroxymethyl resin (0.156 g, 0.1 mmol) as prepared using Method A, peptide **13** was synthesised using in situ neutralisation protocols for Boc SPPS. 0.190 g of dried resin was obtained from the synthesis (91 % yield based on expected dry weight from a 0.1 mmol scale). The peptide-resin was cleaved using TFA/TMSBr for 2 h. Preparative HPLC (15-40 % B in 30 min), yielded the title compound *H*-LYRAI-*MPAA* **13** as a white powder (0.028 g, 28 %) (purity > 98 %).

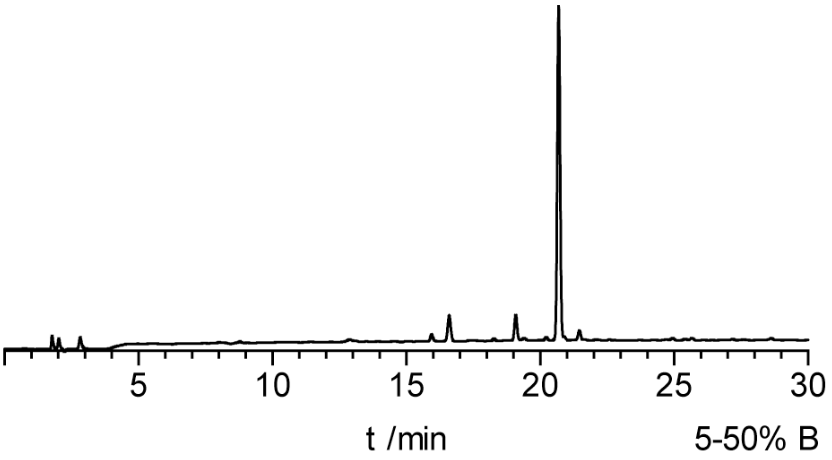

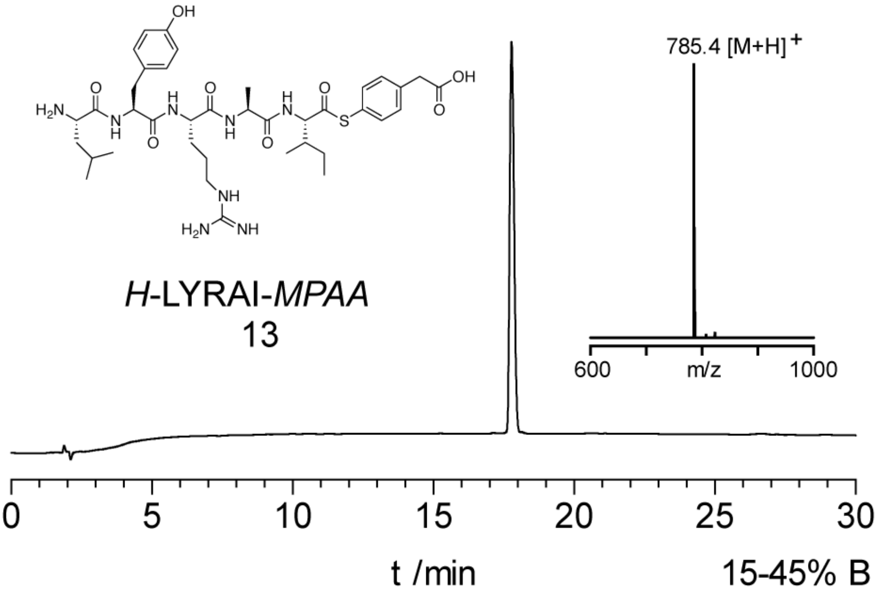

Figure S12. Above: Analytical HPLC of crude peptide *H*-LYRAI-*MPAA* **13**: R_t 20.7 min (5-50 % B over 30 min, λ = 214 nm). Below: Analytical HPLC of pure peptide *H*-LYRAI-*MPAA* **13**: R_t 17.8 min (15-45 % B over 30 min, λ = 214 nm); MALDI-TOF-MS: m/z = 785.4 $[M+H]^+$ (First isotope), calc.: 785.4.

***H*-LYRAL-*MPAA* (14).** Starting with Merrifield hydroxymethyl resin (0.156 g, 0.1 mmol) prepared using Method A, peptide **14** was synthesised using in situ neutralisation protocols for Boc SPPS. 0.185 g of dried resin was obtained from the synthesis (82 % yield based on expected dry weight from a 0.1 mmol scale). The peptide-resin was cleaved using TFA/TMSBr for 2 h. Preparative HPLC (15-40 % B in 30 min), yielded the title compound *H*-LYRAL-*MPAA* **14** as a white powder (0.023 g, 23 %) (purity > 98 %).

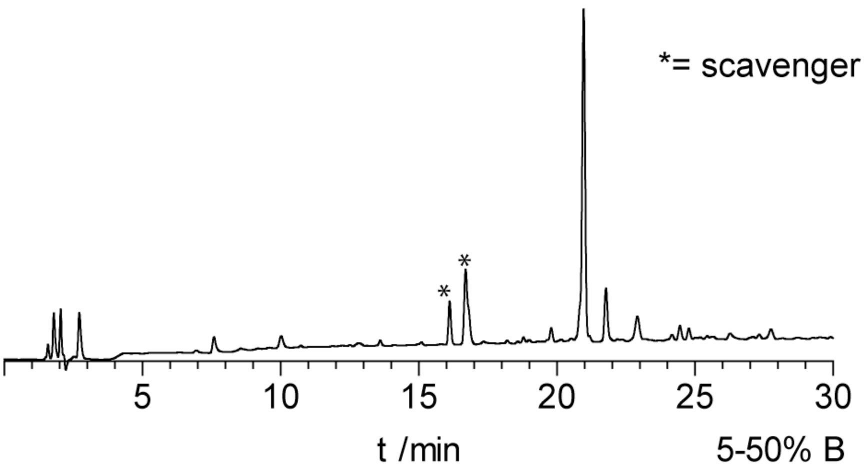

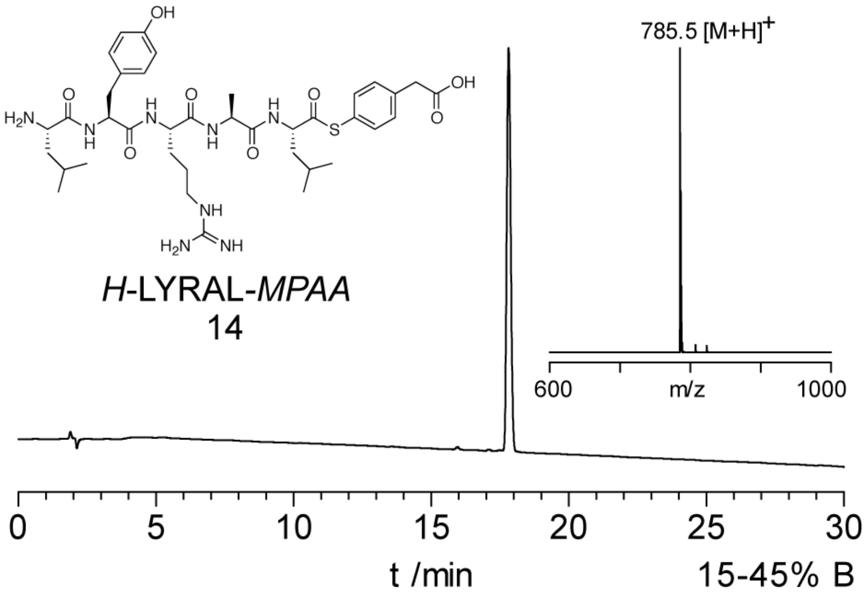

Figure S13. Above: Analytical HPLC of crude peptide *H*-LYRAL-*MPAA* **14**: R_t 20.9 min (5-50 % B over 30 min, λ = 214 nm); Below: Analytical HPLC of pure peptide *H*-LYRAL-*MPAA* **14**: R_t 17.9 min (15-45 % B over 30 min, λ = 214 nm); MALDI-TOF-MS: m/z = 785.5 $[M+H]^+$ (First isotope), calc.: 785.4.

H-LETVSpTQELY-*MPAA* (15). Starting with Merrifield hydroxymethyl resin (0.130 g, 0.1 mmol) prepared using Method A, peptide **15** was synthesised using in situ neutralisation protocols for Boc SPPS. 0.179 g of dried resin was obtained upon completion of the synthesis (57 % yield based on expected dry weight from a 0.1 mmol scale). The peptide-resin was cleaved using TFA/TMSBr/thioanisole/EDT (3/0.6/0.15/0.075 mL: 2 x 5 h). Preparative HPLC (20-45 % B in 30 min), yielded the title compound *H*-LETVSpTQELY-*MPAA* **15** as a white powder (0.011 g, 23 %) (purity > 98 %).

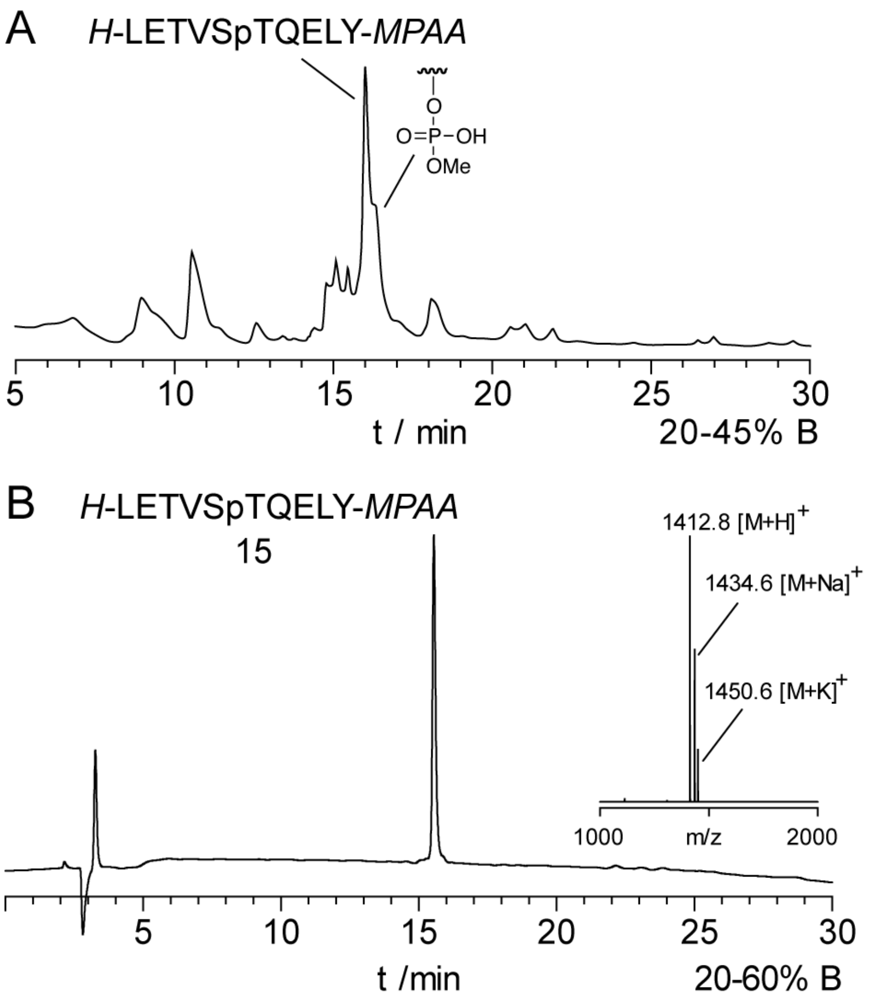

Figure S14. Crude and pure HPLC traces of *H*-LETVS$_p$TQELY-*MPAA* **(15)**. A. Crude HPLC analysis of H-LETVS$_p$TQELY-*MPAA* showed incomplete deprotection of the –OMe from the phosphate using one treatment of TFA/TMSBr/Thioanisole/EDT (3/0.6/0.15/0.075) B. After a second treatment with TFA/TMSBr and purification the title compound was obtained. Analytical HPLC of pure peptide *H*-LETVSpTQELY-*MPAA* **15**: R_t 15.5 min (20-60 % B over 30 min, λ = 214 nm); MALDI-TOF-MS: m/z = 1412.8 $[M+H]^+$, 1434.6 $[M+Na]^+$, 1450.6 $[M+K]^+$ (First isotope), calc.: 1412.6, 1434.6, 1450.5.

***H*-LKAQADIYKA-*MPAA* (16).** Starting with Merrifield hydroxymethyl resin (0.16 g, 0.2 mmol), peptide **16** was prepared using Method B described above and synthesised using in situ neutralisation protocols for Boc SPPS. 0.372 g of dried resin was obtained upon completion of the synthesis (66 % yield based on expected dry weight from a 0.2 mmol scale). A portion of the peptide-resin (0.115 g, 0.06 mmol) was cleaved using TFA/TMSBr for 1.5 h. Preparative HPLC (20-45 % B in 30 min), yielded the title compound *H*-LKAQADIYKA-*MPAA* **16** as a white powder (0.016 g, 24 %) (purity > 98 %).

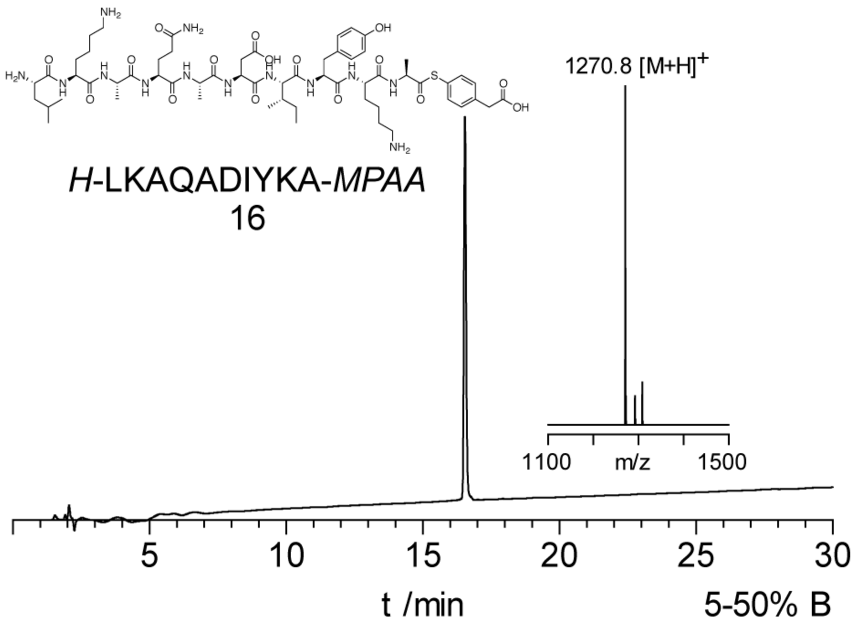

Figure S15. Analytical HPLC of pure peptide *H*-LKAQADIYKA-*MPAA* **16**: R_t 16.6 min (5-50 % B over 30 min, λ = 214 nm); MALDI-TOF-MS: m/z = 1270.8 $[M+H]^+$ (First isotope), calc.: 1270.7.

***H*-dAla-Arg-Arg-Arg-dNal-Arg-Phe(4-F)-dNle-Gln-Trp-Thr-*MPAA* (17).** Starting with Merrifield hydroxymethyl resin (0.272 g, 0.2 mmol) prepared using Method A, peptide **17** was synthesised using in situ neutralisation protocols for Boc SPPS. 0.496 g of dried resin was obtained from the synthesis (84 % yield based on expected dry weight from a 0.2 mmol scale). A portion of the peptide-resin (0.130 g, 0.05 mmol) was cleaved using TFA/TMSBr for 3 h. Preparative HPLC (15-40 % B in 30 min), yielded the title compound *H*-dAla-Arg-Arg-Arg-dNal-Arg-Phe(4-F)-dNle-Gln-Trp-Thr-*MPAA* **17** as white powder (0.016, 16 %) (purity > 98 %).

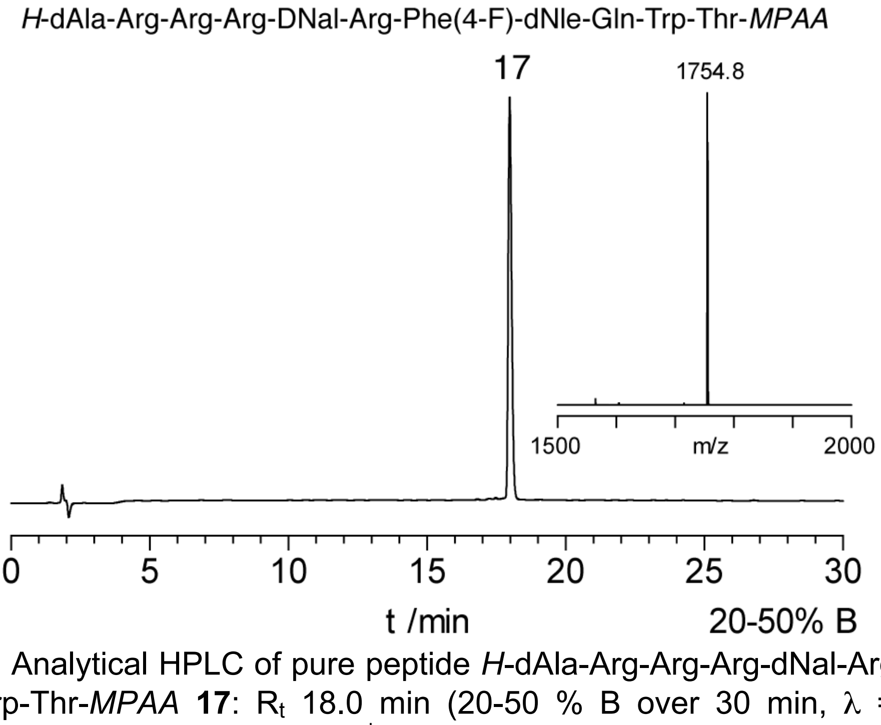

Figure S16. Analytical HPLC of pure peptide *H*-dAla-Arg-Arg-Arg-dNal-Arg-Phe(4-F)-dNle-Gln-Trp-Thr-*MPAA* **17**: R_t 18.0 min (20-50 % B over 30 min, λ = 214 nm); MALDI-TOF-MS: m/z = 1754.8 $[M+H]^+$ (First isotope), calc.: 1754.9.

***H*-CdYVYNTRSGWRWYT-*MPAA* (18).** Starting with Merrifield hydroxymethyl resin (0.133g, 0.1 mmol) prepared using Method B, peptide **18** was synthesised using in situ neutralisation protocols for Boc SPPS. 0.455 g of dried resin was obtained upon completion of the synthesis (88 % yield based on expected dry weight from a 0.1 mmol scale). A portion of the peptide-resin (0.133 g, 0.03 mmol) was cleaved using TFA/TMSBr for 2 h. Preparative HPLC (20-50 % B in 30 min), yielded the title compound *H*-CdYVYNTRSGWRWYT-*MPAA* **18** as white powder (0.019 g, 28 %) (purity > 98 %).

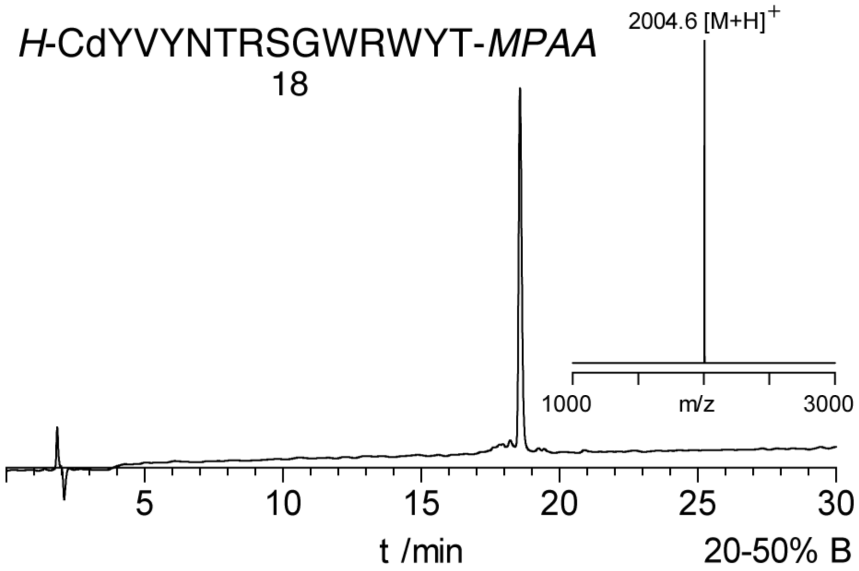

Figure S17. Analytical HPLC of pure peptide *H*-CdYVYNTRSGWRWYT-*MPAA* **18**: R_t 18.7 min (20-50 % B over 30 min, λ = 214 nm); MALDI-TOF-MS: m/z = 2004.6 $[M+H]^+$ (First isotope), calc.: 2004.9.

***H*-AEQH(DNP)KIVMETVPVLKAQADIYKA-*MPAA* (19)**. Starting with Merrifield hydroxymethyl resin (0.19 g, 0.15 mmol) derivatised using Method B, peptide **19** was synthesised using in situ neutralisation protocols for Boc SPPS. 0.368 g of dried resin was obtained upon completion of the synthesis (51 % yield based on expected dry weight from a 0.15 mmol scale). A portion of the peptide-resin (0.104 g, 0.04 mmol) was cleaved using TFA/TMSBr for 2 h. Preparative HPLC (20-50 % B in 30 min), yielded the title compound *H*-AEQH(DNP)KIVMETVPVLKAQADIYKA-*MPAA* **19** as white powder (0.01 g, 14 %) (purity > 98 %).

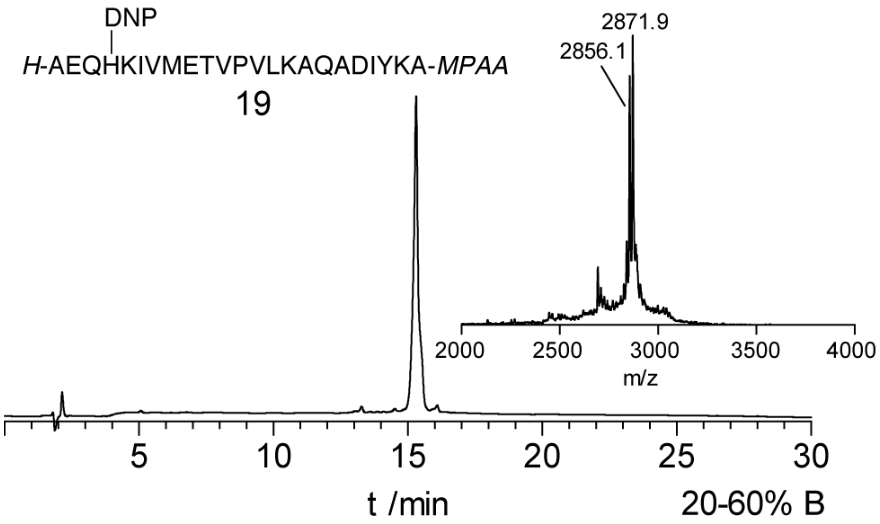

Figure S18. Analytical HPLC of pure peptide *H*-AEQH(DNP)KIVMETVPVLKAQADIYKA-*MPAA* **19**: R_t 15.4 min (20-60% B over 30 min, λ = 214 nm); MALDI-TOF-MS: m/z = 2856.1 $[M+H-NO_2]^+$ (Avg. Mass), calc.: 2855.3.

***H*-LEDLRQQLQQAEEALVAKQELI-*MPAA* (20)**. Starting with Merrifield hydroxymethyl resin (0.19 g, 0.15 mmol) prepared using Method B, peptide **20** was synthesised using in situ neutralisation protocols for Boc SPPS. 0.530 g of dried resin was obtained upon completion of the synthesis (80 % yield based on expected dry weight from a 0.15 mmol scale). A portion of the peptide-resin (0.1 g, 0.03 mmol) was cleaved using TFA/TMSBr for 1.5 h. Preparative HPLC (25-55 % B in 30 min), yielded the title compound *H*-LEDLRQQLQQAEEALVAKQELI-*MPAA* **20** as white powder (0.016 g, 19 %) (purity > 98 %).

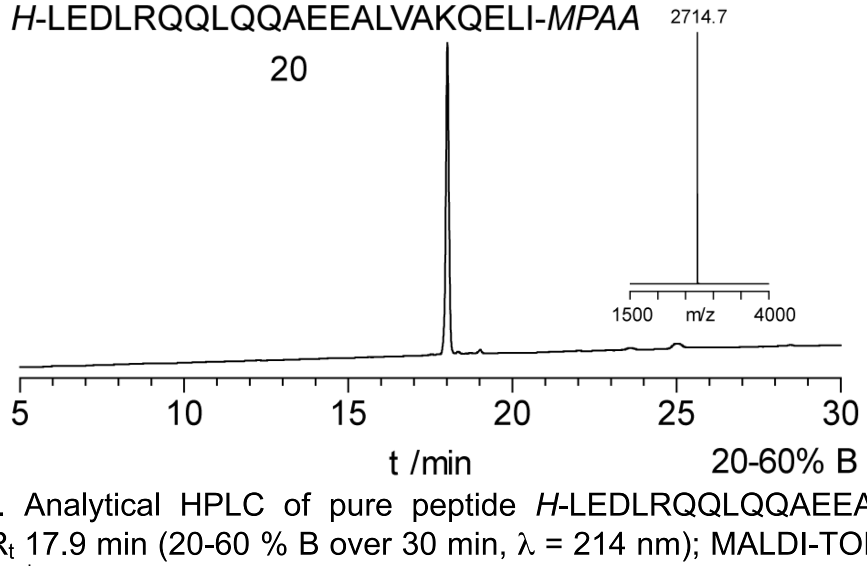

Figure S19. Analytical HPLC of pure peptide *H*-LEDLRQQLQQAEEALVAKQELI-*MPAA* **20**: R_t 17.9 min (20-60 % B over 30 min, λ = 214 nm); MALDI-TOF-MS: m/z = 2714.7 $[M+H]^+$ (First isotope), calc.: 2715.4.

***H*-LEDLRQQLQQAEEALVAKQELIDKL-*MPAA* (21)**. Starting with Merrifield hydroxymethyl resin (0.19 g, 0.15 mmol) prepared using Method B, peptide **21** was synthesised using in situ neutralisation protocols for Boc SPPS. 0.509 g of dried resin was obtained upon completion of the synthesis (68 % yield based on expected dry

weight from a 0.15 mmol scale). A portion of the peptide-resin (0.1 g, 0.03 mmol) was cleaved using TFA/TMSBr for 1.5 h. Preparative HPLC (25-55 % B in 30 min), yielded the title compound *H*-LEDLRQQLQQAEEALVAKQELIDKL-*MPAA* **21** as a white powder (0.014 g, 13 %) (purity > 98 %).

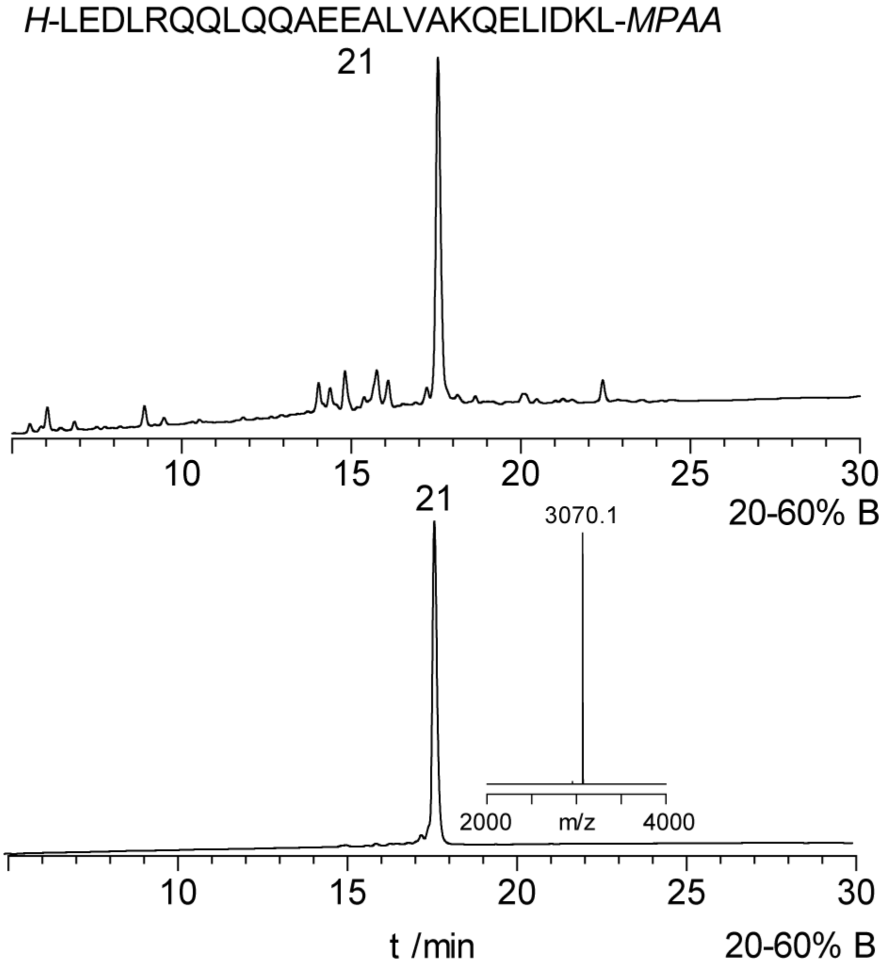

Figure S20. Crude (above) and pure (lower) HPLC traces of LEDLRQQLQQAEEALVAKQELIDKL-*MPAA* **(21)**. Above: Analytical HPLC of crude **21**: R_t 17.6 min (20-60 % B over 30 min, λ = 214 nm) from a single treatment of TFA/TMSBr/Thioanisole/EDT (1/0.05/0.05/0.025) for 1.5 h. Below: Analytical HPLC of pure **21**: R_t 17.6 min (20-60 % B over 30 min, λ = 214 nm); MALDI-TOF-MS: m/z = 3070.1 $[M+H]^+$ (First isotope), calc.: 3070.6.

***H*-LEDLRQQLQQAEEALVAKQELIDKLKEEA-*MPAA* (22)**. Starting with Merrifield hydroxymethyl resin (0.133 g, 0.1 mmol) prepared using Method B, peptide **22** was synthesised using in situ neutralisation protocols for Boc SPPS. 0.373 g of dried resin was obtained upon completion of the synthesis (68 % yield based on expected dry weight from a 0.1 mmol sc le). A portion of the peptide-resin (0.12 g, 0.03 mmol) was cleaved using TFA/TMSBr for 1.5 h. Preparative HPLC (25-55 % B in 30 min), yielded the title compound *H*-LEDLRQQLQQAEEALVAKQELIDKLKEEA-*MPAA* **22** as white powder (0.011 g, 10 % yield) (purity > 98 %).

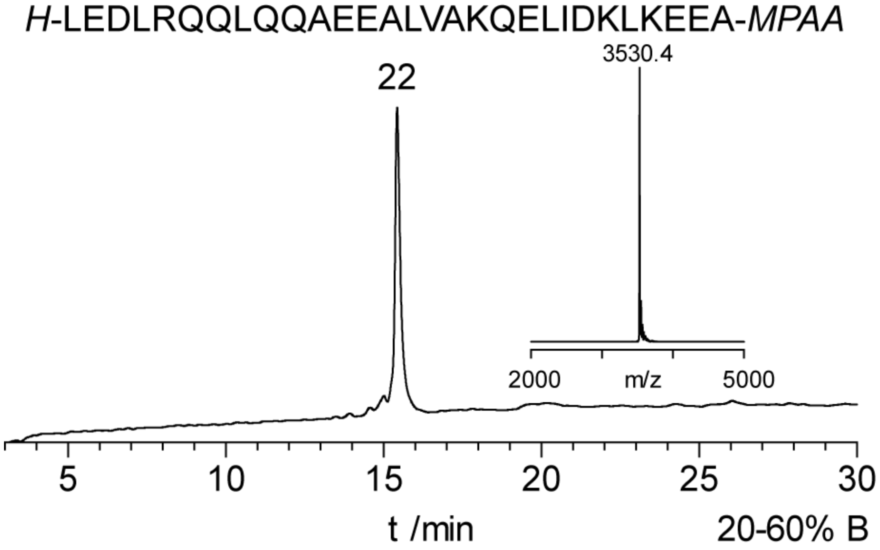

Figure S21. Analytical HPLC of pure *H*-LEDLRQQLQQAEEALVAKQELIDKLKEEA-*MPAA* **22**: R_t 15.4 min (20-60 % B over 30 min, λ = 214 nm); MALDI-TOF-MS: m/z = 3530.4 $[M+H]^+$ (Avg. Mass), calc.: 3531.0.

IV: Peptide Cyclisations

Cyclic CdYVYNTRSGWRWYT (23). Peptide **18** (4 mg, 1.7 mmol; 1 mM) was dissolved in phosphate buffer (1.7 mL, 200 mM NaH_2PO_4, 2 mM EDTA, 6 M Guanidine HCl) with TCEP (0.015 g.mL^{-1}, 50 mM final concentration) and MPAA (0.5 mg.mL^{-1}, 3 mM final concentration). The reaction was allowed to incubate at 40 ˚C and monitored by analytical HPLC by taking aliquots (2 µL) of the reaction mixture and diluting with buffer A (8 µL). The samples were injected on an RP-C18 column using a linear gradient of 20-50 % B in 30 min with a flow rate of 1 mL min^{-1}. After 15 min, the cyclisation was complete by analytical HPLC and diluted with Buffer A. Semi-preparative HPLC (20-50 % B in 30 min with a flow rate of 15 mL min^{-1}) yielded the title compound as a white powder (2.64 mg, 75 %).

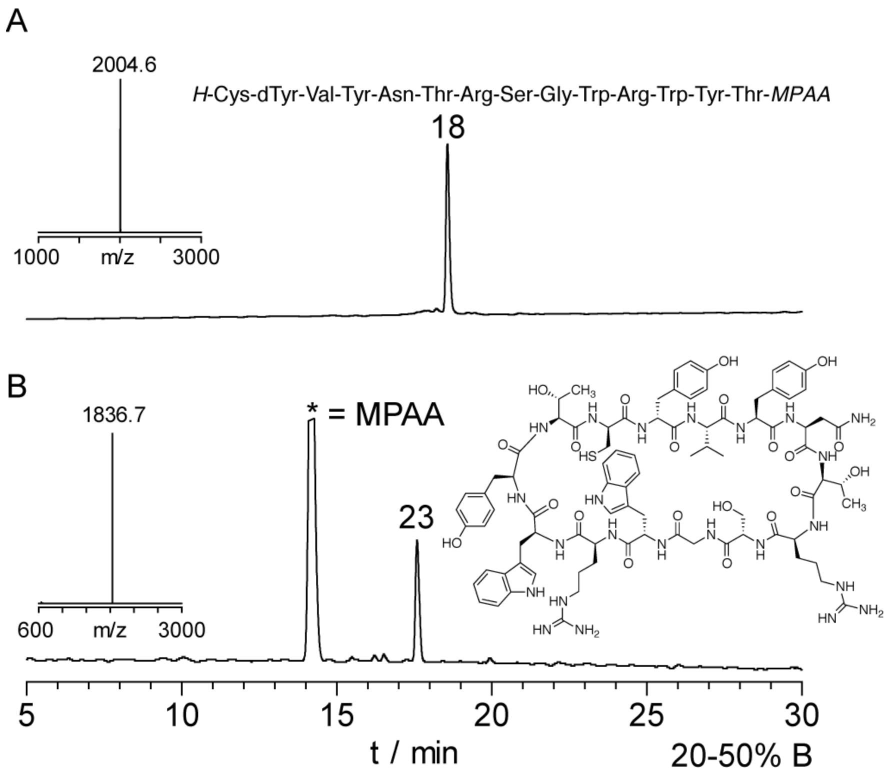

Figure S22. Cyclisation of CdYVYNTRSGWRWYT-*MPAA.* A) HPLC and MALDI-TOF-MS analysis of the purified CdYVYNTRSGWRWYT-*MPAA.* B) HPLC and MALDI-TOF-MS analysis of the cyclisation after 15 min. There is no presence of the starting material. HPLC of **23**: R_t 17.7 min (20-50 % B over 30 min, λ = 214 nm); m/z = 1836.7 $[M+H]^+$ (First isotope), calc.: 1836.8.

Cyclorasin 9A5 (24). The cyclisation of peptide **17** was performed as previously described by Houghten[S6] without purification of the crude starting material. Crude peptide **17** (0.042 g, 0.028 mmol) was dissolved in a mixture of CH_3CN:H_2O (7:1) imidazole (12 mL, final concentration of imidazole 1 M). The reaction was allowed to incubate at 40 ˚C for 2 h and followed by analytical HPLC by taking aliquots (2 µL) of the reaction mixture and diluting with buffer A (8 µL). The samples were injected on an RP-C18 column using a linear gradient of 25-55 % B in 30 min with a flow rate of 1 mL min^{-1}. After 2 h, the cyclisation was determined complete, the reaction mixture was diluted with Buffer A and lyophilized to dryness. The resulting powder was redissolved in Buffer A and purified on a semi-preparative HPLC column (25-55 % B in 30 min) to give the title compound **24** as a white powder (7.4 mg, 13 % overall yield).

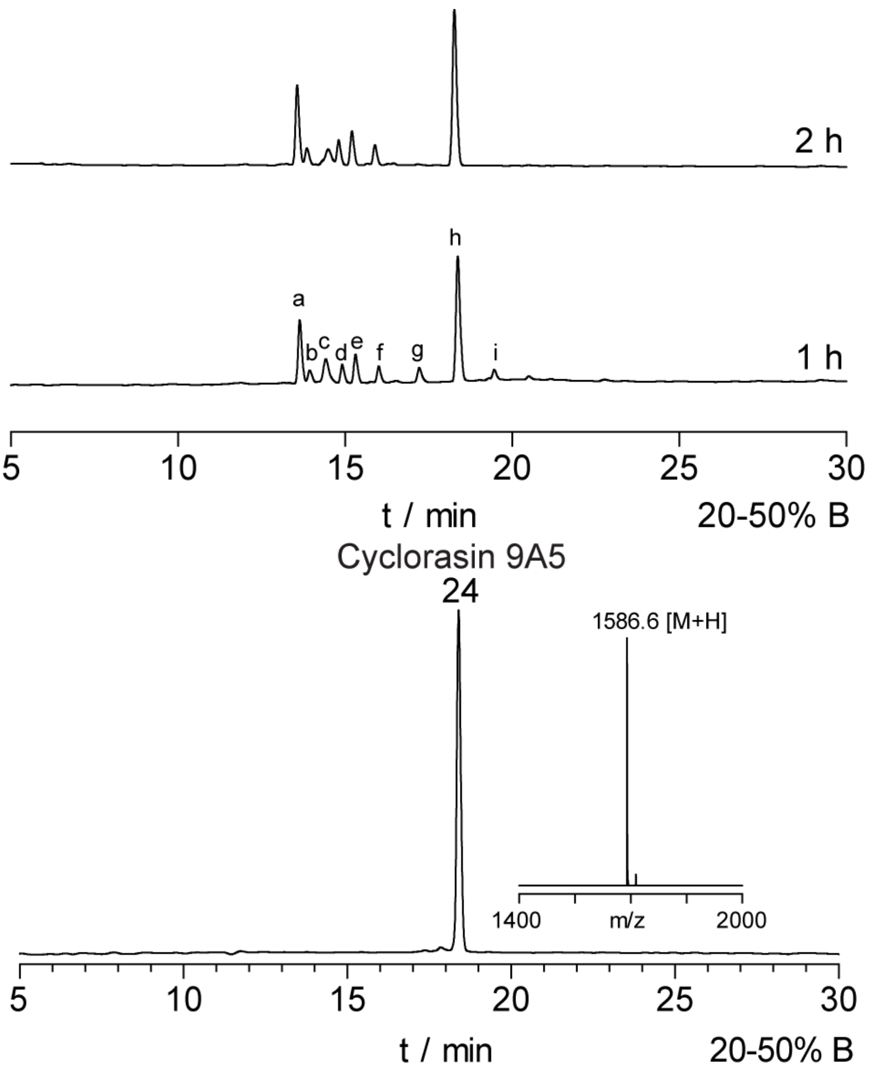

Figure S23. Cyclorasin 9A5 cylisation. Above: Time course of cyclisation of the linear *H*-dAla-Arg-Arg-Arg-dNal-Arg-Phe(4-F)-dNle-Gln-Trp-Thr-*MPAA* to Cyclorasin 9A5 **24**. a) hydrolysis of the peptide thioester b) non head-to-tail cyclised product c) MPAA d,e,f) non head-to-tail cyclised product g) starting material h) correctly cyclised product i) artifact from the column. Below: HPLC of pure Cyclorasin 9A5 **24**: R_t 18.1 min (20-50 % B over 30 min, λ = 214 nm); MALDI-TOF of the main peak: m/z = 1586.6 $[M+H]^+$ (First isotope), calc.: 1586.9.

V. CHK2 phosphopeptide ligation

Cloning, Expression, and Purification:

DNA encoding CHK2 residues 73-538 was amplified from human CHK2 cDNA clone and ligated into pGex-6p1 with a TEV protease site (ENLYFQC) introduced at the N-terminal before residue Ile74. TEV digested CHK2 (73-538) S73C was purified as described.[S7]

Native Chemical Ligation:

The synthetic C-terminal thioester peptide **15** at a concentration of 250 µM was incubated with 100 µM purified CHK2 (73-538) S73C at room temperature in 100 mM phosphate buffer, pH 7.0, 150 mM NaCl, 10 mM TCEP, 75 mM 4-Mercaptophenylacetic acid (MPAA) to form the ligated protein pT68CHK2Lig.

SDS-PAGE:

Samples from Expressed Protein Ligation (EPL) time course studies were run on a Nu-PAGE™ 4-12 % Bis-Tris 1.0mm Gel from Life Technologies in 1x Nu-PAGE™ MES SDS Running Buffer. An XCell-Surelock™ mini-cell electrophoresis system was used to run the above gel at 200 Volts for 45 min. The gel was stained with InstantBlue™ from expedeon.

Gel-filtration profile:

Gel-filtration profiles of unligated (CHK2(73-538) S73C) and ligated (pT68CHK2Lig) were obtained using Akta Purifier (GE Healthcare Life Science). Samples were applied to a Superdex S200 10/300 GL column equilibrated in 20 mM Tris-HCl, 150 mM NaCl, 0.5 mM TCEP, pH 8.0 at a flow rate of 0.5 mL min^{-1} (Figure 3D).

VI. Biological analysis of Cyclorasin 9A5

Protein Expression and Purification material and methods:

Human wt-K-Ras and oncogenic K-Ras_G12V proteins (amino acids 1-185) were cloned in pGEX-6P-1 vector that encodes a glutathione S-transferase (GST) tag at the N-terminal of the expressed protein. The constructs were transformed in *Escherichia coli* BL21 cells – the cells were grown in Luria Broth (LB) media in the presence of 0.1 mg mL^{-1} ampicillin at 37 °C to an OD_{700} then induced by the addition of 0.1 mM IPTG. The bacterial culture was then incubated overnight at 18 °C and harvested the second day by centrifugation. The cell pellet was incubated at 4 °C in lysis buffer (50 mM Tris-HCL, 150 mM NaCl, 0.5 mM TCEP, pH 7.5) in the presence of protease inhibitor cocktail tablets (Sigma) for 30 min followed a sonication step. The cell lysate were centrifuged and the supernatant were incubated with glutathione-Sepharose 4B beads (GE Healthcare) for 1 h at 4 °C. The beads were washed with washing buffer (50 mM Tris-HCL, 150 mM NaCl, 0.5 mM TCEP, pH 7.5). The beads were re-suspended in 15 mL washing buffer and proteins were cleaved of the beads by the addition of HRV 3C protease (in-house made) and incubated overnight at 4 °C. The beads were centrifuged and the supernatant containing the cleaved proteins were concentrated to 300 µL followed by gel-filtration as a second purification step. The protein fractions were collected, concentrated and snap frozen in liquid nitrogen then stored at -80 °C.

Preparation of K-Ras_GDP and K-Ras_GTPγS:

Both Human wt-K-Ras and oncogenic K-Ras_G12V proteins where incubated with the nucleotide exchange buffer (20 mM Tris-HCl, 1 mM EDTA, 25 mM NaCl, pH 7.4) containing either 1 mM GDP or GTPγS and incubated overnight at 4 °C. 5 mM $MgCl_2$ was added the following day and proteins were incubated for further 15 min on ice. Proteins were then purified by gel-filtration, concentrated and snap frozen in liquid nitrogen then stored at -80 °C.

Microscale thermophoresis (MST):

Microscale thermophoresis (MST) measurements were performed using a NanoTemper Monolith™ NT.115 instrument (NanoTemper Technologies GmbH, München, Germany). Protein samples were labelled with the amine reactive dye NT-647 using the Monolith™ NT.115 Protein Labeling Kit RED-NHS. Labeling levels (generally in the range 0.3-0.4 dye molecules per protein) were determined using calculated extinction coefficients and e647 = 250,000 $M^{-1}cm^{-1}$ for the dye concentration. In a typical experiment 20 µL aliquots of a 100 nM stock solution of labelled protein were mixed with 20 µL aliquots of a serial dilution of binding partner. These solutions were then loaded into standard treated capillaries and MST measurements were performed at 25 °C using 20-40 % LED power and 40-60 % IR-Laser power. The laser Laser-On time was 30 s and Laser-Off time 5 s. All measurements were performed at least 3 times.

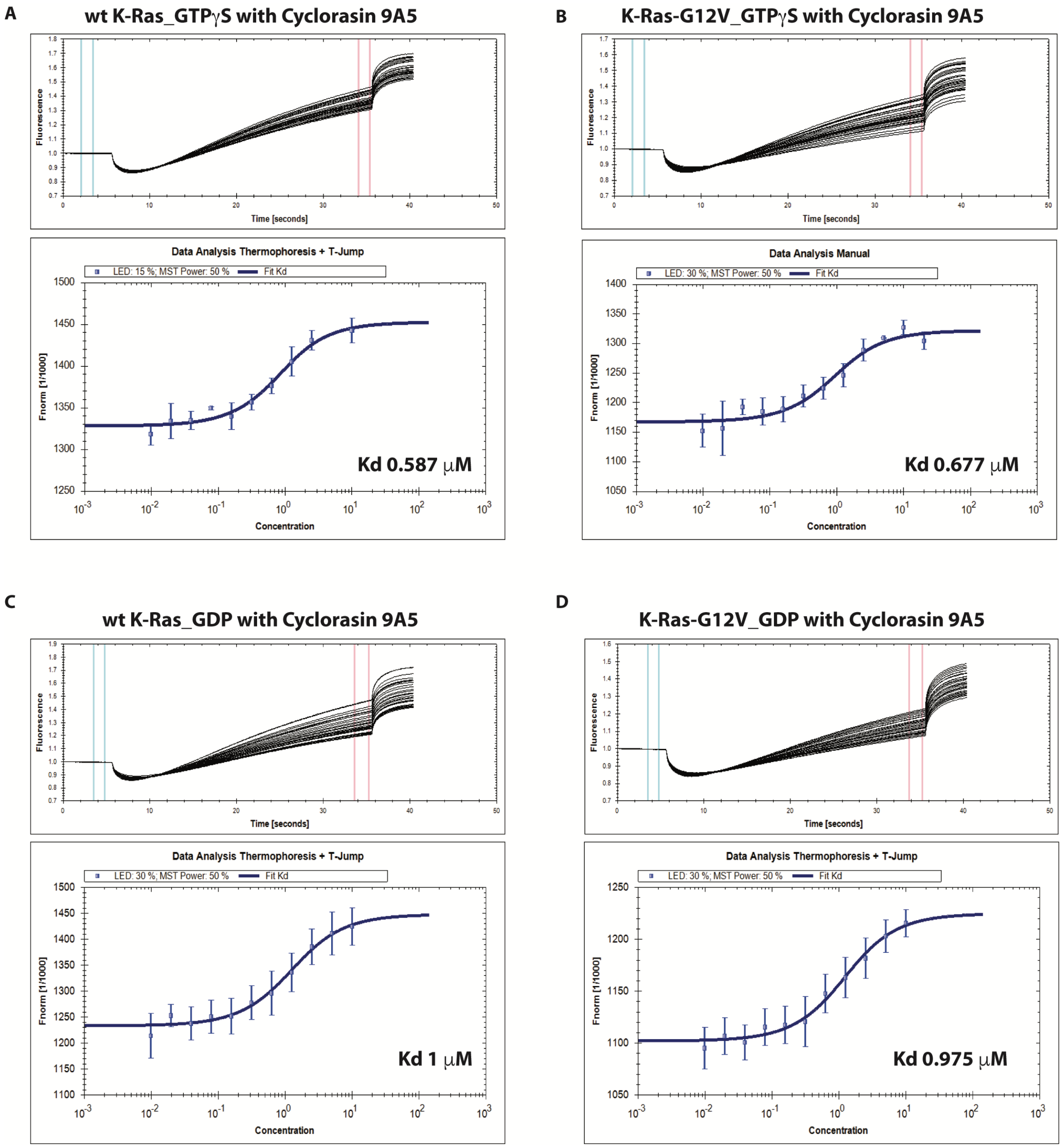

Figure S24. Microscale thermophoresis data showing the binding affinity of the Cyclorasin 9A5 with wt-K-Ras_GTPγS (A), wt-K-Ras_GDP (C), K-Ras-G12V_ GTPγS (B) and K-Ras-G12V_ GDP (D). The Kd values for each interaction were calculated as an average of three measurements.

VII. References

[S1] N. Fujii, A. Otaka, N. Sugiyama, M. Hatano, H. Yajima, *Chem. Pharm. Bull.* **1987**, *35*, 3880-3883.

[S2] T. M. Hackeng, J. H. Griffin, P. E. Dawson, *Proc. Natl. Acad. Sci. USA* **1999**, *96*, 10068-10073.

[S3] D. Bang, B. L. Pentelute, Z. P. Gates, S. B. Kent, *Org. Lett.* **2006**, *8*, 1049-1052.

[S4] R. J. Topping, I. Nuiry, J. Mastriona, J. A. Moss, *Tetrahedron Lett.* **2008**, *49*, 2907-2910.

[S5] M. Schnolzer, P. Alewood, A. Jones, D. Alewood, S. B. H. Kent, *Int. J. Pept. Protein Res.* **1992**, *40*, 180-193.

[S6] Y. M. Li, A. Yongye, M. Giulianotti, K. Martinez-Mayorga, Y. P. Yu, R. A. Houghten, *J. Comb. Chem.* **2009**, *11*, 1066-1072.

[S7] J. Li, I. A. Taylor, J. Lloyd, J. A. Clapperton, S. Howell, D. MacMillan, S. J. Smerdon, *J. Biol. Chem.* **2008**, *283*, 36019-36030.
